# 3D Quantitative Ablation Margins for Prediction of Ablation Site Recurrence After Stereotactic Image-Guided Microwave Ablation of Colorectal Liver Metastases: A Multicenter Study

**DOI:** 10.3389/fonc.2021.757167

**Published:** 2021-11-15

**Authors:** Simeon J. S. Ruiter, Pascale Tinguely, Iwan Paolucci, Jennie Engstrand, Daniel Candinas, Stefan Weber, Robbert J. de Haas, Koert P. de Jong, Jacob Freedman

**Affiliations:** ^1^ Department of Hepato-Pancreato-Biliary Surgery and Liver Transplantation, University Medical Center Groningen, University of Groningen, Groningen, Netherlands; ^2^ Division of Surgery, Department of Clinical Sciences, Karolinska Institutet at Danderyd Hospital, Stockholm, Sweden; ^3^ Department of Visceral Surgery and Medicine, Inselspital, University Hospital Bern, Bern, Switzerland; ^4^ ARTORG Center for Biomedical Engineering Research, University of Bern, Bern, Switzerland; ^5^ Department of Radiology, University Medical Center Groningen, University of Groningen, Groningen, Netherlands

**Keywords:** liver neoplasm, ablation techniques, computer-assisted therapies, stereotactic techniques, interventional radiology, margin assessment

## Abstract

**Background:**

Three-dimensional (3D) volumetric ablation margin assessment after thermal ablation of liver tumors using software has been described, but its predictive value on treatment efficacy when accounting for other factors known to correlate ablation site recurrence (ASR) remains unknown.

**Purpose:**

To investigate 3D quantitative ablation margins (3D-QAMs) as an algorithm to predict ASR within 1 year after stereotactic microwave ablation (SMWA) for colorectal liver metastases (CRLM).

**Materials and Methods:**

Sixty-five tumors in 47 patients from a prospective multicenter study of patients undergoing SMWA for CRLM were included in this retrospective 3D-QAM analysis. Using a previously developed algorithm, 3D-QAM defined as the distribution of tumor to ablation surface distances was assessed in co-registered pre- and post-ablation CT scans. The discriminatory power and optimal cutoff values for 3D-QAM were assessed using receiver operating characteristic (ROC) curves. Multivariable logistic regression analysis using generalized estimating equations was applied to investigate the impact of various 3D-QAM outputs on 1-year ASR while accounting for other known influencing factors.

**Results:**

Ten of the 65 (15.4%) tumors included for 3D-QAM analysis developed ASR. ROC analyses identified i) 3D-QAM <1 mm for >23% of the tumor surface, ii) 3D-QAM <5 mm for >45%, and iii) the minimal ablation margin (MAM) as the 3D-QAM outputs with optimal discriminatory qualities. The multivariable regression model without 3D-QAM yielded tumor diameter and *KRAS* mutation as 1-year ASR predictors. When adding 3D-QAM, this factor became the main predictor of 1-year ASR [odds ratio (OR) 21.67 (CI 2.48, 165.21) if defined as >23% <1 mm; OR 0.52 (CI 0.29, 0.95) if defined as MAM].

**Conclusions:**

3D-QAM allows objectifiable and standardized assessment of tumor coverage by the ablation zone after SMWA. Our data shows that 3D-QAM represents the most important factor predicting ASR within 1 year after SMWA of CRLM.

## Introduction

Thermal ablation such as microwave ablation (MWA) is a tissue-sparing treatment alternative to hepatic resection in selected patients, yielding similar treatment efficacy, recurrence-free survival, and overall survival as described in non-randomized studies (1–5). Treatment efficacy can be significantly enhanced by using stereotactic navigation technology for precise targeting of liver tumors as opposed to conventional targeting techniques (6, 7).

The main remaining drawback of thermal ablation is the relatively frequent suspicion of viable tumor tissue at the edge of the ablation zone [ablation site recurrence (ASR)] reported between 2% and 41% for colorectal liver metastases (CRLM) (8, 9). Several factors negatively correlated with ASR after thermal ablation of CRLM have been identified in multivariable models, including tumor size (10–15), prior liver resection (16), carcinoembryonic antigen (CEA) levels (17), proximity to larger intrahepatic vessels (10, 13, 18), and mutational status of *KRAS* oncogenes (16, 19–21). Most importantly, the completeness of ablation as described by the extent of the ablation margin represents an independent predictor of ASR (16, 18, 19, 22, 23). Taking margin evaluation one step further, ablation margins assessed in 3D using dedicated software have been described and applied for prediction of ASR (24, 25). However, the correlation of volumetric ablation margins with ASR, while taking into account other previously described independent predictors of ASR, has not yet been investigated (16, 19–21). Moreover, measurement accuracy of previous studies investigating volumetric ablation margins as a predictive factor of ASR was limited to a slice thickness of computed tomography (CT) images of 3–5 mm (24, 25).

The aim of the present study was to acquire further knowledge on predictors of ASR after stereotactic microwave ablation (SMWA) of CRLM by i) investigating three-dimensional quantitative ablation margin (3D-QAM) as a predictor of 1-year ASR, considering factors previously shown to be negatively correlated with ASR, and ii) identifying and comparing different outputs of 3D-QAM and their effect sizes in a multivariable model in data from a prospective European multicenter trial.

## Materials and Methods

### Study Population

**Figure 1:**
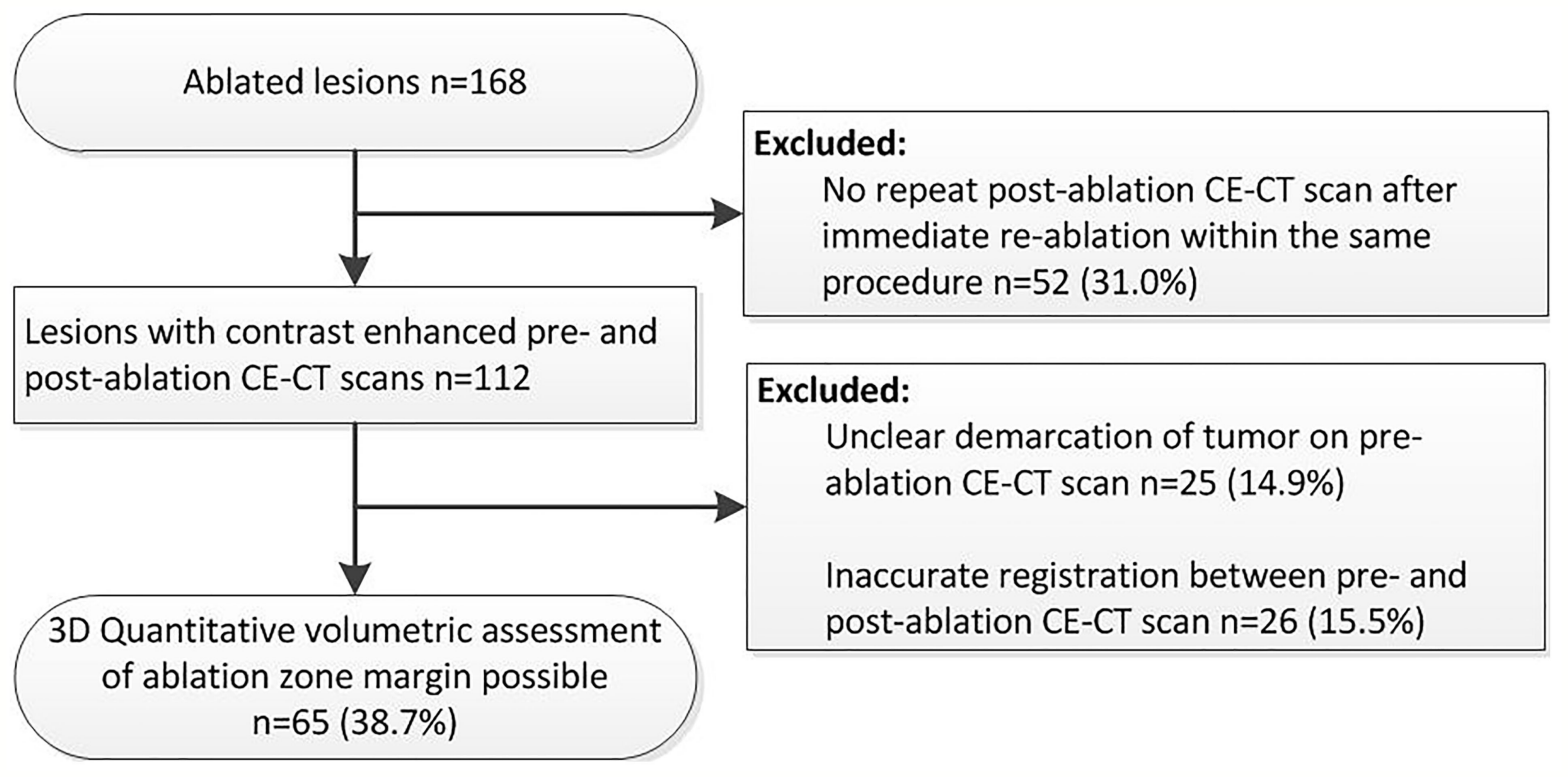
Flowchart of retrospective inclusion of tumors for three-dimensional quantitative ablation margin (3D-QAM) analysis.

Retrospective analysis of prospectively collected data from patients included in the MAVERRIC (Microwave Ablation *Versus* Resection for Resectable Colorectal liver metastases) trial was performed. The MAVERRIC trial is a prospective cohort study comparing SMWA to surgical resection for resectable CRLM performed in three European tertiary hepato-pancreato-biliary surgery centers (Inselspital, Bern, Switzerland; University Medical Center Groningen, Groningen, Netherlands; and Danderyd Hospital, Stockholm, Sweden). Patients had a maximum of five CRLM ≤30 mm, considered eligible for both resection and SMWA as decided in local multidisciplinary tumor board meetings, and deliberately underwent SMWA. The trial was registered at clinicaltrials.gov (NCT02642185) and approved by all regional ethical review boards. While the primary endpoint of the MAVERRIC study is overall survival, the current paper focuses on factors influencing ASR within 1 year. Between January 2016 and December 2018, 98 patients were included in the MAVERRIC study and a total of 168 CRLM treated with SMWA. Of these, 65 tumors in 47 patients were eligible for enrollment in the current retrospective 3D-QAM analysis (**Figure 1**).

### Stereotactic Microwave Ablation Procedure and Follow-Up

All procedures were performed in the interventional CT suite by interdisciplinary teams consisting of specifically trained interventional radiologists and hepatobiliary surgeons with large experience in image-guided tumor ablation (all operators have performed >100 SMWA procedures). Patients were placed under general anesthesia and positioned on a vacuum mattress to eliminate patient movement. High-frequency jet ventilation (26) or controlled apnea was applied during image acquisition and antenna manipulations. Procedures were performed with 64-multidetector row CT systems (Siemens Somatom Sensation 64; Toshiba Aquilion One, Toshiba). All contrast-enhanced (CE)-CT scans had an in-plane resolution of 0.6–1.0 mm × 0.6–1.0 mm and a slice thickness of maximum 1 mm. The CAS-ONE system (Cascination AG, Bern, Switzerland) or the Needle Positioning System (NPS; DEMCON Advanced Mechatronics, Enschede, Netherlands) was used for stereotactic tumor targeting. Workflows were described in detail previously (27–29). A CE-CT scan performed in portal venous phase was acquired for planning of ablation trajectories before stereotactic placement of ablation antennas. A non-enhanced CT scan was acquired after each antenna manipulation for validation of antenna positions. Single-needle MWA was performed with Acculis (Angiodynamics, Latham, NY, USA), Amica (HS Hospital Service SPA, Rome, Italy), or Emprint (Covidien, Minneapolis, MN, USA) systems. A CE-CT scan was performed in portal venous phase directly after antenna extraction, and validation of technical success was evaluated visually by direct overlay of co-registered pre- and post-ablation images. Technical success at the time of the procedure was defined as complete coverage of the tumor by the ablation zone aiming for an ablation margin of ideally 5–10 mm. If this was not achieved, immediate re-ablation with SMWA was performed. If after immediate re-ablation no final CE-CT scan could be performed due to intravenous contrast dosing limits, these patients were not eligible for inclusion in the current retrospective analysis. The first follow-up imaging (MRI or CT) was performed within 3 months after the intervention and thereafter every 3–4 months during the first year, applying standardized terminology and reporting criteria (30). ASR was defined as the appearance of tumor foci at the edge of ablation zone during follow-up (31).

### Data Extraction and Definitions

Patient and tumor characteristics, details on neoadjuvant chemotherapy, CEA levels, and follow-up data on ASR within 1 year from SMWA treatment were extracted from the prospective secured MAVERRIC study database. *KRAS* mutational status was assessed on specimen of the primary tumor. A subcapsular tumor location was defined as a minimal distance of the edge of the tumor to the liver capsule of ≤5 mm. A perivascular location was defined as a minimal distance of ≤5 mm from the edge of the tumor to a vessel with a diameter of ≥3 mm.

### Quantitative Ablation Margin and Ablation Site Recurrence

**Figure 2:**
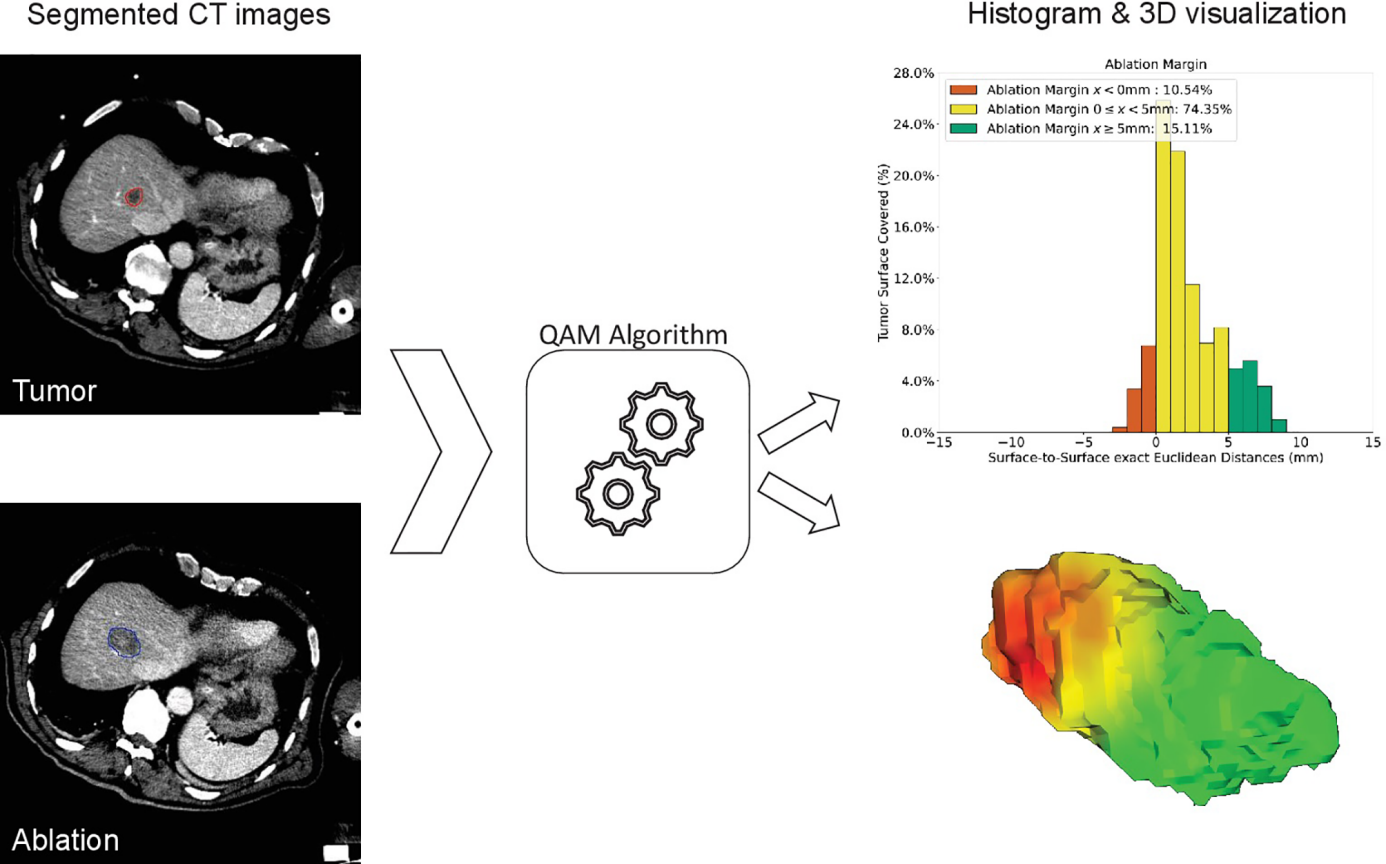
Method of computation of three-dimensional quantitative ablation margin (3D-QAM). Left: segmentation of tumor (red) and ablation (blue) volumes. After calculating 3D-QAM, defined as the distribution of tumor to ablation surface distance (32), the QAM output can be displayed as a histogram (upper right image) or as a 3D visualization of the ablation volume surface (lower right image). In the histogram, each bin represents the percentage of tumor surface covered by a specific distance (e.g., 1 mm). Margins to the tumor surface of >5 mm are marked in green, of 0–5 mm in yellow, and of <0 mm (residual tumor) in red. In this example, 15.11% of the tumor surface was covered by an ablation zone of at least 5 mm (green).

Ablation margins were assessed retrospectively using a previously developed algorithm for 3D-QAM computation based on a signed Euclidean surface distance map, including an algorithm to account for subcapsular tumors. To calculate 3D-QAM, tumor and ablation volumes were segmented on co-registered pre- and post-ablation scans using a semiautomatic segmentation tool (Amira 6.3, ThermoFisher Scientific, USA), which at the time of procedures was not integrated in the navigation software. 3D-QAM was defined as the distribution of surface to surface distances between the tumor and the ablation boundaries, as shown in **Figure 2**. The mathematical model and open-source code of the algorithm were described in detail previously (32). Output of the 3D-QAM algorithm included numerical values and visual illustrations of relative distributions of ablation margin distances, i.e., the total tumor surface area covered by the ablation margins.

A regression model of factors influencing ASR within 1 year was created (baseline model A), including factors previously described to be associated with ASR, as assessed in a meticulous literature search (13–22). To assess the optimal application of 3D-QAM and its influence on ASR, 3D-QAM was then added in varying outputs to the baseline model as follows: i) Model B: All clinical factors of model A plus 3D-QAM as a continuous variable of the minimal ablation margin (MAM) (33); ii) Model C: All clinical factors of model A plus 3D-QAM as the optimal discrimination threshold of all possible 3D-QAM distributions, as identified in receiver operating characteristic (ROC) curves; and iii) Model D: All clinical factors of model A plus 3D-QAM as the optimal discrimination threshold of the previously described 5-mm margin (34). Cutoff values identified in the ROC curves using the Youden index (maximum sensitivity/specificity) were used to define binary variables for models C and D.

### Statistical Analyses

Means and standard deviation or median and interquartile range (IQR) were reported for continuous variables and number and percentage for categorical variables. The chi-square test was applied to compare categorical variables and Mann–Whitney *U* test for nonparametric continuous variables. Multivariable logistic regression analysis was applied to investigate factors associated with 1-year ASR. All variables previously reported and thought to potentially affect ASR after ablation of CRLM were included as independent variables (baseline model A). Consecutively, 3D-QAM was added in various outputs as described above (models B, C, and D). Repeated-measures analysis using generalized estimating equations (GEEs) was applied to account for intra-class correlations, arising due to ablation of multiple tumors in the same patient and inclusion of patient-specific variables (CEA levels, *KRAS* mutation) while focusing on a tumor-specific outcome (ASR). An independent correlation structure and a robust estimator of covariance were applied. Effect estimates are reported as odds ratios (ORs) and 95% confidence intervals (CIs). The threshold for statistical significance was set at p < 0.05. Comparisons between regression models were performed using ANOVA. R (R Core Team, 2019) and RStudio (RStudio Inc., USA) were used for statistical analyses.

## Results

### Clinical and Intervention-Related Data

**Table 1** shows clinicopathological characteristics of the 47 patients with 65 tumors at the time of SMWA. Median tumor diameter was 13 mm (IQR 10–20 mm), with two tumors treated with a diameter above 30 mm (34 and 41 mm) due to tumor growth between date of patient inclusion and date of SMWA. Sixteen (34.0%) patients received neoadjuvant chemotherapy before ablation, with no significant differences between centers (p = 0.955). All ablations were performed with a single antenna with a median power of 100 W (IQR 80–120 W) and median time of 4 min (IQR 3–8 min) per tumor. Median number of antenna positions was 1 (IQR 1–5) per ablation, and median dose length product was 1,192 mGy * cm (IQR 960–1,925 mGy * cm) per procedure. All tumors included in this study were presumed to be treated technically successful, with no residual tumors detected at the end of the SMWA procedure, as assessed by direct overlay of co-registered pre- and post-ablation images.

**Table 1:** Clinicopathological characteristics.

Patient characteristics	Total patients (n = 47)
Sex (male:female)	32:15
Age in years at intervention^†^	69 (62–74)
Type of liver metastases	
Synchronous	18 (38.3)
Metachronous	23 (48.9)
Recurrence after partial hepatectomy	6 (12.8)
Neoadjuvant chemotherapy prior to ablation	16 (34.0)
CEA level before ablation (μg/L)^†^	3.6 (1.9–7.8)
Treated liver metastases per patient^†^	2 (1–2)
**Primary tumor characteristics**	**Total patients (n = 47)**
Primary tumor location	
Right colon	16 (34.0)
Left colon and rectum	31 (66.0)
Nodal status	
N0	18 (38.3)
N1–N2	29 (61.7)
KRAS mutational status	
mutated	22 (46.8)
wild type	21 (44.7)
missing	4 (8.5)
**Liver metastases characteristics**	**Total tumors (n = 65)**
Location	
Left-sided liver	13 (20.0)
Right-sided liver	50 (76.9)
Segment 1	2 (3.1)
Subcapsular (<5 mm)	30 (46.2)
Perivascular (<5 mm of >3-mm-sized vessel)	16 (24.6)
Diameter (mm) at intervention^†^	13 (10–20)

Unless otherwise specified, data are shown as number and percentages.

^†^Data are shown as medians with interquartile ranges in parentheses.

CEA, carcinoembryonic antigen.

### Quantitative Ablation Margins and Ablation Site Recurrence

**Figure 3:**
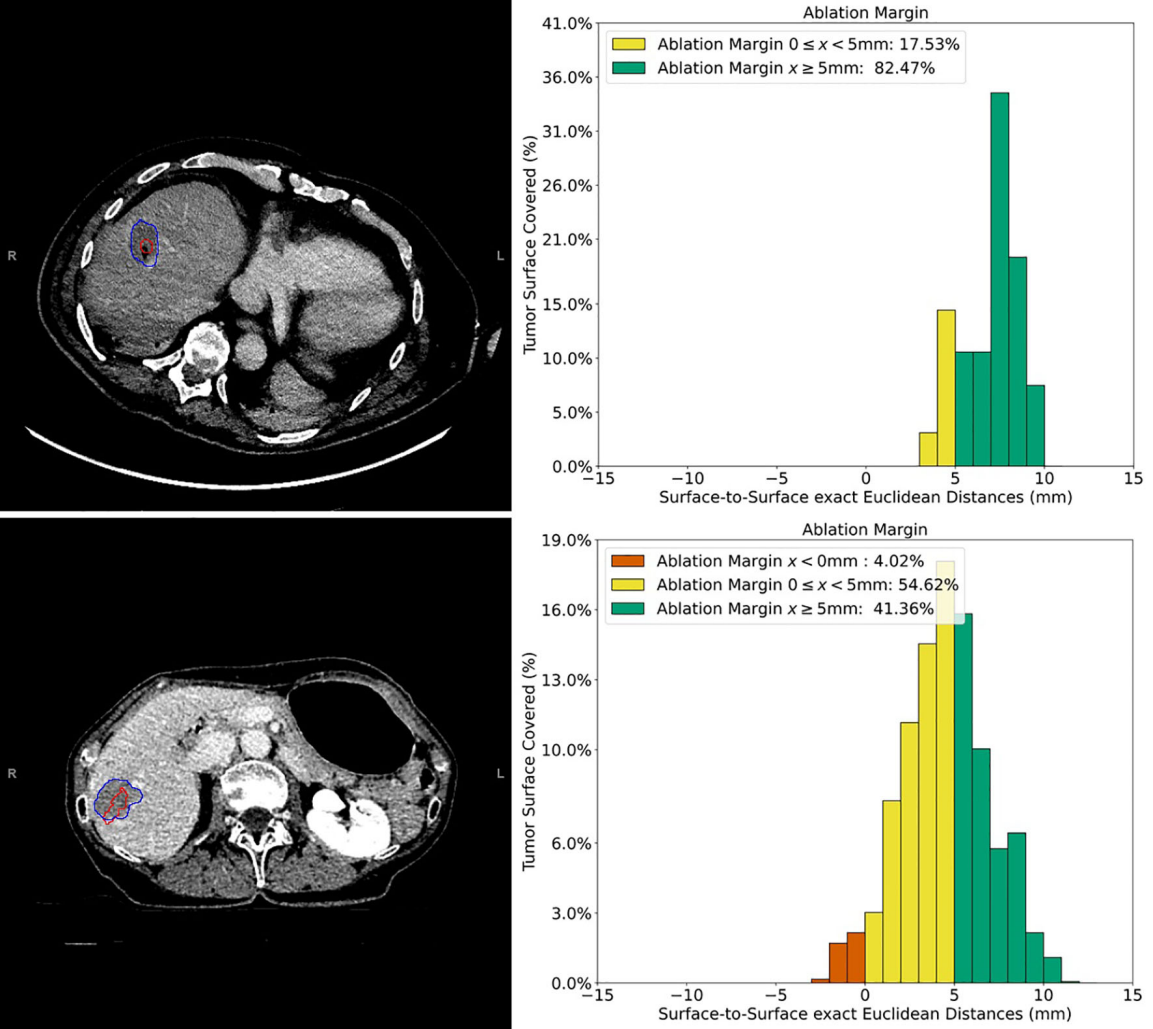
Example cases of three-dimensional quantitative ablation margin (3D-QAM) assessment in patients with colorectal liver metastases (CRLMs). Upper images: contrast-enhanced CT image of a CRLM in segment 8 treated with stereotactic microwave ablation (SMWA), with corresponding histogram of 3D-QAM on the right. Lower images: contrast-enhanced CT image of CRLM in segment 7 and its corresponding histogram of 3D-QAM distribution on the right. Segmentations of the tumor in both CT scans are marked in red, and the corresponding ablation volumes are marked in blue. Relative distribution of ablation margins with respect to percentage of tumor surface covered is displayed as a histogram, colored green for margins >5 mm, yellow for margins 0–5 mm, and red for residual tumor (margins <0 mm).

**Figure 4:**
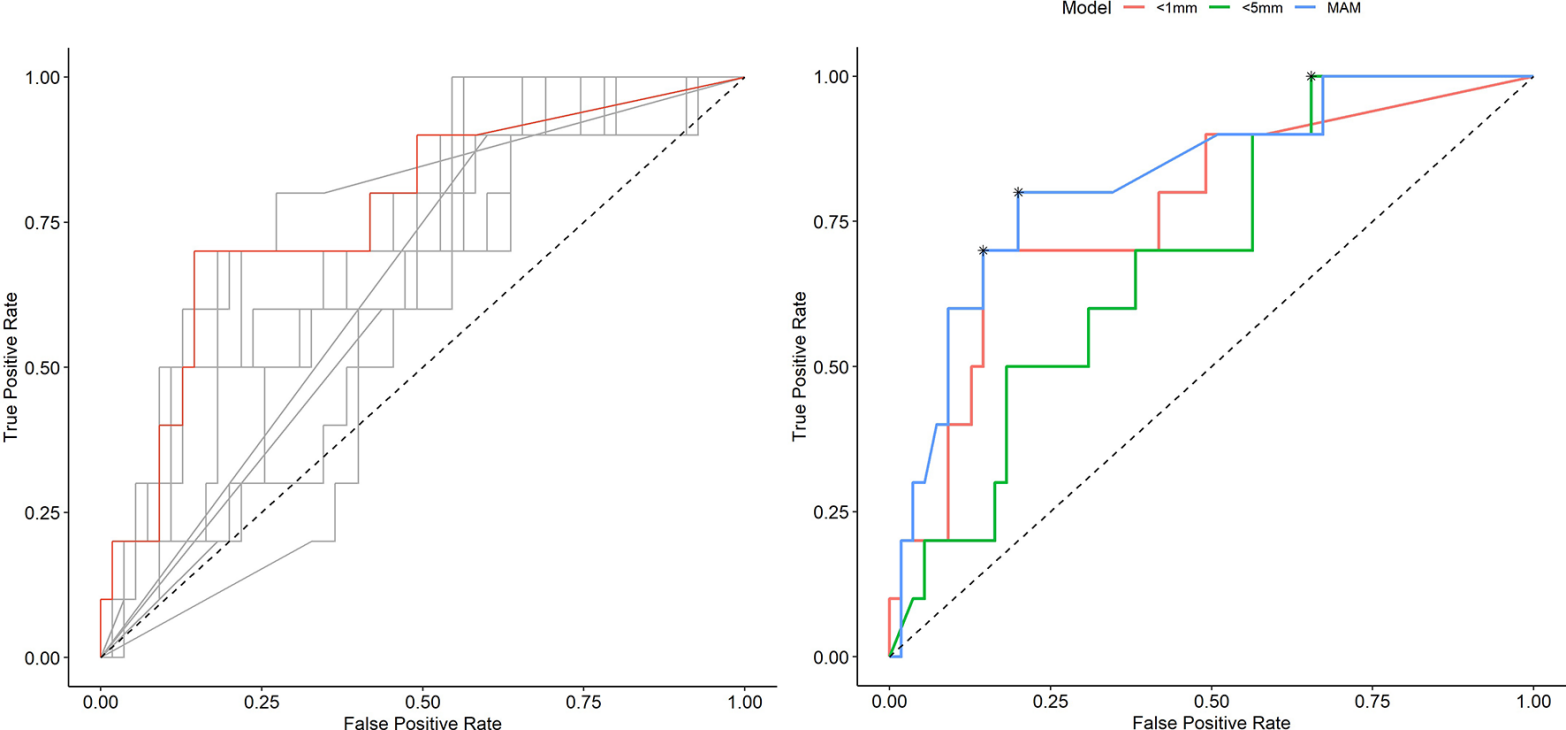
Receiver operating characteristic (ROC) curves illustrating the diagnostic ability of varying outputs of three-dimensional quantitative ablation margins (3D-QAMs). Left: All ROC curves with percentages of tumor coverage of least 0, 1, 2, 3, 4, 5, 6, 7, 8, 9, or 10 mm. The highest accuracy [area under the curve (AUC) = 0.77] in predicting ablation site recurrence (ASR) was found for the tumor coverage of 1 mm indicated in red. Right: Blue curve, minimal ablation margin (MAM); red curve, percentages of tumor coverage <1 mm; green curve, percentages of tumor coverage <5 mm. Optimal discrimination thresholds of each curve are indicated with an asterisk (*).

**Table 2** shows numerical results for 3D-QAM analyses. Two examples of tumors treated with SMWA with corresponding 3D-QAM assessment and histograms are shown in **Figure 3**. Ten of the 65 included tumors (15.4%) developed ASR at follow-up within 1 year, with no differences in ASR between the three centers (p = 0.356). Smaller MAM (p = 0.001) and higher percentages of margins <0 mm in 3D-QAM (p = 0.003) were seen in tumors with ASR compared to tumors without ASR. No ASR occurred in tumors with coverage of 100% of the tumor surface by an ablation margin of at least 2 mm (100% 3D-QAM of ≥2 mm) and 90% coverage of at least 3 mm (90% 3D-QAM of ≥3 mm). The ROC curves investigating the diagnostic ability of varying 3D-QAM outputs are shown in **Figure 4**. Of all percentages of 3D-QAM analyzed between 0 and 10 mm, the area under the curve (AUC) was greatest (AUC = 0.77) for a 3D-QAM of 1 mm; hence, the distribution of 3D-QAM <1 mm was chosen for model C. The ROC curves for 3D-QAM <5 mm (AUC = 0.69) and 3D-QAM <1 mm yielded optimal discrimination thresholds at 45% and 23%, respectively (**Figure 4**).

**Table 2:** Numerical results of 3D-QAM assessment.

	ASR	No ASR	p-value
Minimum margin (mm)	-2.4 (-3.2 to -1.4)	0.0 (-0.9 to 2.4)	0.001
Median margin (mm)	2.7 (1.8–4.5)	4.6 (3.5–5.7)	0.021
Maximum margin (mm)	8.8 (8.4–9.3)	9.3 (7.6–10.6)	0.495
3D-QAM <0 mm (%)	11.5 (1.9–18.9)	0.0 (0.0–0.9)	0.003

Unless otherwise specified, data are shown as medians and interquartile ranges.

ASR, ablation site recurrence; 3D-QAM, three-dimensional quantitative ablation margin.

Results from the logistic regression analysis investigating factors associated with ASR are summarized in **Table 3**. The baseline model without inclusion of 3D-QAM (model A) yielded tumor diameter [OR 1.11 (CI 1.05, 1.18)] and *KRAS* mutation [OR 0.29 (CI 0.09, 0.97)] as statistically significant factors affecting ASR. When adding 3D-QAM defined as MAM as a continuous variable (model B), only 3D-QAM remained significant [OR 0.52 (CI 0.29, 0.95; p = 0.03)]. When adding 3D-QAM defined as a categorical binary variable with the identified cutoff of 23% of tumor coverage with a margin of <1 mm (model C), again, 3D-QAM had the largest effect on ASR [OR 21.67 (CI 2.84, 165.21; p = 0.003)]. Accordingly, the comparison of models B and C with baseline model A yielded significant differences between the models (p = 0.003 and p = 0.03). Model D using a 3D-QAM with identified cutoff of >45% tumor coverage with a margin of <5 mm was not interpretable, since no event of ASR occurred in the group of <45% tumor coverage with a margin of <5 mm. This resulted in a numerical error, rendering statistical analysis of model D impossible. The entire statistical analyses are available in **Supplementary Material 1**.

**Table 3:** Multivariable logistic regression analyses (GEE) of factors potentially predicting ASR per tumor using varying outputs of 3D-QAMs.

	Model A Baseline		Model B MAM (continuous variable)		Model C 3D-QAM >23% <1 mm (y/n)	
	OR (95% CI)	p-value	OR (95% CI)	p-value	OR (95% CI)	p-value
Neoadjuvant chemotherapy (y/n)	1.22 (0.33, 4.54)	0.766	4.14 (0.52, 33.18)	0.181	1.97 (0.35, 11.04)	0.439
Previous resection (y/n)	0.12 (0.01, 1.29)	0.080	0.30 (0, 461.69)	0.748	0.79 (0.01, 74.51)	0.921
Perivascular location (<5 mm of >3-mm-sized vessel) (y/n)	1.89 (0.52, 6.85)	0.334	1.94 (0.23, 16.53)	0.544	1.49 (0.15, 14.45)	0.730
KRAS mutation (y/n)	0.29 (0.09, 0.97)	0.044	0.47 (0.07, 3.4)	0.455	0.62 (0.08, 4.61)	0.641
CEA level (continuous variable)	1.01 (1, 1.02)	0.192	1.02 (0.86, 1.2)	0.858	1.01 (0.87, 1.17)	0.886
Tumor diameter (mm, continuous variable)	1.11 (1.05, 1.18)	< 0.001	1.10 (0.98, 1.25)	0.116	1.13 (1.01, 1.27)	0.035
3D-QAM algorithm	–	–	0.52 (0.29, 0.95)	0.034	21.67 (2.84, 165.21)	0.003

Model A: Basic model without including 3D-QAM; Model B: MAM as a continuous variable of; Model C: Binary model with cutoff of >23% covered surface with a margin of 3D-QAM <1 mm.

OR, odds ratio; CI, confidence interval; CEA, carcinoembryonic antigen; MAM, minimal ablation margin; 3D-QAM, three-dimensional quantitative ablation margin; ASR, ablation site recurrence; GEE, generalized estimating equation.

## Discussion

This work investigated the application of novel 3D-QAM algorithm (32) as a tool to analyze ablation margins and its impact on short-term local tumor control in a quantitative and objectifiable manner. After analyzing various statistical models with and without including 3D-QAM, we demonstrated that 3D-QAM defined as >23% <1 mm was the main independent factor predicting ASR within 1 year. To our knowledge, this is the first study investigating the effect size of quantitative volumetric ablation margins on ASR in consideration of other factors known to influence ASR development, such as *KRAS* mutational status.

Ablation margin assessment after thermal ablation of hepatic malignancies has been explored before, with most studies suggesting a prognostic value of MAM smaller than 5 mm with regard to ASR (16, 18, 19, 22, 35). While MAM evaluations in these studies were mainly performed by evaluation of pre- and post-ablation CT images in 2D (34), the additional value of 3D-QAM has been reported (24, 25). Laimer et al. (25) assessed percentages of predetermined safety margins in 3D and found a 100% 3D ablation margin of 3 mm and a 90% 3D ablation margin of 6 mm to be indicators for ASR. These results confirmed the importance and potential value of 3D-QAM, however not accounting for other previously described risk factors for ASR such as *KRAS* mutational status. In the present study, we deliberately included all factors previously shown to be negatively associated with ASR in multivariable models. For 3D-QAM defined as the MAM as a continuous variable (model B), the odds to develop ASR within 1 year was on average 48% lower for every additional millimeter of MAM. In model C, a <1-mm margin of more than 23% of the tumor surface yielded on average a 21 times larger odds to develop ASR. While CIs for both QAM outputs were large probably due to the low event rates of ASR, 3D-QAM defined as >23% <1 mm represented the main factor influencing ASR, with all other variables becoming less significant after adding 3D-QAM to the baseline model. As opposed to previous studies identifying 5–10-mm margins as relevant regarding ASR (33), the significantly smaller ablation margins identified in this study suggest that a technology-driven volumetric margin assessment allows a more detailed and objectifiable reporting as opposed to a visual assessment.

The strengths of the proposed 3D-QAM algorithm using surface distance mapping include a description of ablation margins in subfractions of relative distributions of ablation margins relative to the total tumor surface. Aiming toward the most detailed possible assessment of margins, the discrimination between, e.g., a 5% *vs*. 20% tumor surface area covered by a certain ablation margin is of crucial importance. While the optimal cutoff of 23% of <1 mm as found in our data might seem complex and unlikely to be reproducible on a wider scale, such volumetric ablation margin assessments will always necessitate the application of dedicated image analysis software, and the integration of what might seem a complex definition would not represent a relevant additional step. An important factor regarding the accuracy of ablation margin assessment in general is the direct dependence on available slice thickness of CE-CT images and on registration errors between pre- and post-ablation images. In the present study, all intra-procedural CE-CT scans were obtained with a maximum slice thickness of 1 mm and all patient motion was minimized, reducing potential inaccuracies of 3D-QAM calculations. Previously described margin assessment tools using slice thickness of 3 mm and up to 5 mm (24, 25) might negatively affect the registration error and accuracy in margin assessment of these tools. The 3D-QAM algorithm will further allow to correlate the area in 3D of actual ASR to the area of MAMs, as opposed to ASR due to satellite lesions (36). The applied 3D-QAM algorithm further allowed inclusion of subcapsular tumors (32), representing more than half of the tumors ablated in this series. This is essential when aiming to apply such novel margin assessment tools on a broad scale. Integrating 3D-QAM assessment into dedicated software protocols, such as the stereotactic navigation technology applied in this study, will allow an objectifiable assessment of completeness of ablation with the potential of immediate re-ablation of these areas, aiming toward a 100% technical success rate in one treatment session. It further enables a true distinction between incomplete ablation and ASR, which would allow further standardization of the reporting of outcomes after ablation. These potentially significant advantages of 3D-QAM underline the use of CT with integrated novel technical solutions for thermal ablation, if available.

The overall ASR rate of 15.4% reported in this paper was in line with other local tumor control rates after thermal ablation of CRLM in selected populations (37). It is well known and was confirmed in baseline model A and model C that tumor size is significantly correlated with ASR development (10–15), which might be overcome by applying SMWA with multiple antennas (22, 34). Other factors previously described to influence ASR were not significant in our model, such as neoadjuvant chemotherapy or *KRAS* mutational status. This might be explained by the shorter follow-up period in this paper as opposed to previous studies showing influences of biological parameters such as *KRAS* mutational status on ASR (19, 20).

A limitation of this study is the differences in follow-up regimens between centers, with one center performing the first follow-up imaging 1 week after ablation and the other centers 3 months after ablation. Despite these differences in follow-up protocols, no significant differences in ASR were observed between the participating centers. Also, distribution of ASR between included and excluded tumors did not significantly differ despite the high exclusion rate from the final ASR model, underlining plausibility of the analysis. Finally, even though patients were included prospectively for the MAVERRIC study, 3D-QAM was assessed retrospectively, and a prospective validation of this algorithm in future clinical research with a larger sample size is needed to confirm the current results.

In conclusion, this study confirmed utility of the 3D-QAM algorithm for assessment of ablation completeness after SMWA of CRLM. The 3D-QAM defined as 23% of <1-mm margin represented the most important predictor of ASR within 1 year, leaving most previously described factors less crucial in our model. If integrated into standard workflows of stereotactic ablation, 3D-QAM might allow to accurately and objectively determine areas of insufficient ablation and immediate retreatment of such, potentially reducing the rate of incomplete ablations and ASR. It might further facilitate objective reporting and standardization of post-ablation follow-up definitions in the future.

## Data Availability Statement

The raw data supporting the conclusions of this article will be made available by the authors without undue reservation.

## Ethics Statement

The ethics committees of all three participating centers (Inselspital, Bern, Switzerland; University Medical Center Groningen, Groningen, Netherlands; and Danderyd Hospital, Stockholm, Sweden) have approved this study. The patients/participants provided their written informed consent to participate in this study.

## Author Contributions

Guarantor of integrity of entire study, JF. Study concepts/study design or data acquisition or data analysis/interpretation, all authors. Article drafting or article revision for important intellectual content, all authors. Agrees to ensure any questions related to the work are appropriately resolved, all authors. Literature research, SR and PT. Data collection, SR, PT, IP, and JF. Statistical analysis, SR, PT, IP, KJ, and JF. Article editing, all authors. All authors contributed to the article and approved the submitted version.

## Funding

This work was partially funded by the Prof. Dr. Max Cloëtta Foundation (to PT), the Swiss Cancer League (to PT), funds from Region Stockholm (ALF to JF and clinical postdoctoral appointment to JE). Funding institutions had no contribution in the design or execution of this project.

## Conflict of Interest

SW and DC are co-founders and shareholders of CAScination AG, manufacturer of one of the navigation systems used for SMWA in this study.

The remaining authors declare that the research was conducted in the absence of any commercial or financial relationships that could be construed as a potential conflict of interest.

## Publisher’s Note

All claims expressed in this article are solely those of the authors and do not necessarily represent those of their affiliated organizations, or those of the publisher, the editors and the reviewers. Any product that may be evaluated in this article, or claim that may be made by its manufacturer, is not guaranteed or endorsed by the publisher.

## References

[1] Hof J Wertenbroek MWJLAE Peeters PMJG Widder J Sieders E de Jong KP. Outcomes After Resection and/or Radiofrequency Ablation for Recurrence After Treatment of Colorectal Liver Metastases. Br J Surg (2016) 103(8):1055–62. doi: 10.1002/bjs.10162 27193207

[2] Tanis E Nordlinger B Mauer M Sorbye H van Coevorden F Gruenberger T. Local Recurrence Rates After Radiofrequency Ablation or Resection of Colorectal Liver Metastases. Analysis of the European Organisation for Research and Treatment of Cancer 40004 and 40983. Eur J Cancer (2014) 50(5):912–9. doi: 10.1016/j.ejca.2013.12.008 24411080

[3] Tinguely P Dal G Bottai M Nilsson H Freedman J Engstrand J. Microwave Ablation *Versus* Resection for Colorectal Cancer Liver Metastases – A Propensity Score Analysis From a Population-Based Nationwide Registry. Eur J Surg Oncol (2020) 46(3):476–85. doi: 10.1016/j.ejso.2019.12.002 31837931

[4] Puijk RS Ruarus AH Vroomen LGPH van Tilborg AAJM Scheffer HJ Nielsen K. Colorectal Liver Metastases: Surgery *Versus* Thermal Ablation (COLLISION) - A Phase III Single-Blind Prospective Randomized Controlled Trial. BMC Cancer (2018) 18(1):1–13. doi: 10.1186/s12885-018-4716-8 30111304 PMC6094448

[5] Van Cutsem E Cervantes A Adam R Sobrero A Van Krieken JH Aderka D. ESMO Consensus Guidelines for the Management of Patients With Metastatic Colorectal Cancer. Ann Oncol (2016) 27(8):1386–422. doi: 10.1093/annonc/mdw235 27380959

[6] Schaible J Pregler B Verloh N Einspieler I Bäumler W Zeman F. Improvement of the Primary Efficacy of Microwave Ablation of Malignant Liver Tumors by Using a Robotic Navigation System. Radiol Oncol (2020) 54(3):295–300. doi: 10.2478/raon-2020-0033 32463387 PMC7409605

[7] Tinguely P Paolucci I Ruiter SJS Weber S de Jong KP Candinas D. Stereotactic and Robotic Minimally Invasive Thermal Ablation of Malignant Liver Tumours - A Systematic Review and Meta-Analysis. Front Oncol (2021) 11:713685. doi: 10.3389/fonc.2021.713685 34631539 PMC8495244

[8] Meijerink MR Puijk RS van Tilborg AAJM Henningsen KH Fernandez LG Neyt M. Radiofrequency and Microwave Ablation Compared to Systemic Chemotherapy and to Partial Hepatectomy in the Treatment of Colorectal Liver Metastases: A Systematic Review and Meta-Analysis. Cardiovasc Intervent Radiol (2018) 41(8):1189–204. doi: 10.1007/s00270-018-1959-3 PMC6021475 29666906

[9] Sotirchos VS Petrovic LM Gönen M Klimstra DS Do RKG Petre EN. Colorectal Cancer Liver Metastases: Biopsy of the Ablation Zone and Margins Can Be Used to Predict Oncologic Outcome. Radiol (2016) 280(3):949–59. doi: 10.1148/radiol.2016151005 PMC5006720 27010254

[10] Leung U Kuk D D’Angelica MI Kingham TP Allen PJ DeMatteo RP. Long-Term Outcomes Following Microwave Ablation for Liver Malignancies. Br J Surg (2015) 102(1):85–91. doi: 10.1002/bjs.9649 25296639 PMC4593505

[11] Groeschl RT Pilgrim CHC Hanna EM Simo KA Swan RZ Sindram D. Microwave Ablation for Hepatic Malignancies. Ann Surg (2014) 259(6):1195–200. doi: 10.1097/SLA.0000000000000234 24096760

[12] Mulier S Ni Y Jamart J Ruers T Marchal G Michel L. Local Recurrence After Hepatic Radiofrequency Coagulation. Ann Surg (2005) 242(2):158–71. doi: 10.1097/01.sla.0000171032.99149.fe PMC1357720 16041205

[13] Urbonas T Anderson EM Gordon-Weeks AN Kabir SI Soonawalla Z Silva MA. Factors Predicting Ablation Site Recurrence Following Percutaneous Microwave Ablation of Colorectal Hepatic Metastases. HPB (2019) 21(9):1175–84. doi: 10.1016/j.hpb.2019.01.007 30777696

[14] Facciorusso A Del Prete V Crucinio N Serviddio G Vendemiale G Muscatiello N. Lymphocyte-To-Monocyte Ratio Predicts Survival After Radiofrequency Ablation for Colorectal Liver Metastases. World J Gastroenterol (2016) 22(16):4211. doi: 10.3748/wjg.v22.i16.4211 27122671 PMC4837438

[15] Takahashi H Kahramangil B Kose E Berber E. A Comparison of Microwave Thermosphere *Versus* Radiofrequency Thermal Ablation in the Treatment of Colorectal Liver Metastases. HPB (2018) 20(12):1157–62. doi: 10.1016/j.hpb.2018.05.012 29929785

[16] Odisio BC Yamashita S Huang SY Kopetz SE Ahrar K Mizuno T. Impact of Prior Hepatectomy History on Local Tumor Progression After Percutaneous Ablation of Colorectal Liver Metastases. J Vasc Interv Radiol (2018) 29(3):395–403. doi: 10.1016/j.jvir.2017.10.026 29395898 PMC5818310

[17] Peng S Huang P Yu H Wen Y Luo Y Wang X. Prognostic Value of Carcinoembryonic Antigen Level in Patients With Colorectal Cancer Liver Metastasis Treated With Percutaneous Microwave Ablation Under Ultrasound Guidance. Med (Baltimore) (2018) 97(10):e0044. doi: 10.1097/MD.0000000000010044 PMC5882454 29517661

[18] Shady W Petre EN Do KG Gonen M Yarmohammadi H Brown KT. Percutaneous Microwave *Versus* Radiofrequency Ablation of Colorectal Liver Metastases: Ablation With Clear Margins (A0) Provides the Best Local Tumor Control. J Vasc Interv Radiol (2018) 29(2):268–75. doi: 10.1016/j.jvir.2017.08.021 PMC5803367 29203394

[19] Odisio BC Yamashita S Huang SY Harmoush S Kopetz SE Ahrar K. Local Tumour Progression After Percutaneous Ablation of Colorectal Liver Metastases According to RAS Mutation Status. Br J Surg (2017) 104(6):760–8. doi: 10.1002/bjs.10490 PMC5391264 28240361

[20] Calandri M Yamashita S Gazzera C Fonio P Veltri A Bustreo S. Ablation of Colorectal Liver Metastasis: Interaction of Ablation Margins and RAS Mutation Profiling on Local Tumour Progression-Free Survival. Eur Radiol (2018) 28(7):2727–34. doi: 10.1007/s00330-017-5273-2 29417253

[21] Jiang B Yan K Zhang Z Yang W Wu W Yin S. The Value of KRAS Gene Status in Predicting Local Tumor Progression of Colorectal Liver Metastases Following Radiofrequency Ablation. Int J Hyperth (2019) 36(1):210–8. doi: 10.1080/02656736.2018.1556818 30663903

[22] Shady W Petre EN Vakiani E Ziv E Gonen M Brown KT. Kras Mutation Is a Marker of Worse Oncologic Outcomes After Percutaneous Radiofrequency Ablation of Colorectal Liver Metastases. Oncotarget (2017) 8(39):66117–27. doi: 10.18632/oncotarget.19806 PMC5630397 29029497

[23] Shady W Petre EN Gonen M Erinjeri JP Brown KT Covey AM. Percutaneous Radiofrequency Ablation of Colorectal Cancer Liver Metastases: Factors Affecting Outcomes—A 10-Year Experience at a Single Center. Radiol (2016) 278(2):601–11. doi: 10.1148/radiol.2015142489 PMC4734163 26267832

[24] Kaye EA Cornelis FH Petre EN Tyagi N Shady W Shi W. Volumetric 3D Assessment of Ablation Zones After Thermal Ablation of Colorectal Liver Metastases to Improve Prediction of Local Tumor Progression. Eur Radiol (2019) 29(5):2698–705. doi: 10.1007/s00330-018-5809-0 PMC6719337 30402706

[25] Laimer G Jaschke N Schullian P Putzer D Eberle G Solbiati M. Volumetric Assessment of the Periablational Safety Margin After Thermal Ablation of Colorectal Liver Metastases. Eur Radiol (2021) 31:6489–99. doi: 10.1007/s00330-020-07579-x PMC8379110 33447860

[26] Galmén K Freedman J Toporek G. Clinical Application of High Frequency Jet Ventilation in Stereotactic Liver Ablations – A Methodological Study [Version 2; Peer Review: 2 Approved]. F1000Research (2018) 7:773. doi: 10.12688/f1000research.14873.2 30271582 PMC6113879

[27] Arnolli MM Buijze M Franken M de Jong KP Brouwer DM Broeders IAMJ. System for CT-Guided Needle Placement in the Thorax and Abdomen: A Design for Clinical Acceptability, Applicability and Usability. Int J Med Robot Comput Assist Surg (2018) 14(1):e1877. doi: 10.1002/rcs.1877 29205787

[28] Heerink WJ Ruiter SJS Pennings JP Lansdorp B Vliegenthart R Oudkerk M. Robotic *Versus* Freehand Needle Positioning in CT-Guided Ablation of Liver Tumors: A Randomized Controlled Trial. Radiology (2019) 290(3):826–32. doi: 10.1148/radiol.2018181698 30667337

[29] Tinguely P Frehner L Lachenmayer A Banz V Weber S Candinas D. Stereotactic Image-Guided Microwave Ablation for Malignant Liver Tumors—A Multivariable Accuracy and Efficacy Analysis. Front Oncol (2020) 10. doi: 10.3389/fonc.2020.00842/full PMC7298123 32587826

[30] Ahmed M. Image-Guided Tumor Ablation: Standardization of Terminology and Reporting Criteria—A 10-Year Update: Supplement to the Consensus Document. J Vasc Interv Radiol (2014) 25(11):1706–8. doi: 10.1148/radiol.14132958 25442133

[31] Kele PG van der Jagt EJ Krabbe PFM de Jong KP. Lack of Anatomical Concordance Between Preablation and Postablation CT Images: A Risk Factor Related to Ablation Site Recurrence. Int J Hepatol (2012) 2012:1–9. doi: 10.1155/2012/870306 PMC3540787 23320184

[32] Sandu R-M Paolucci I Ruiter SJS Sznitman R de Jong KP Freedman J. Volumetric Quantitative Ablation Margins for Assessment of Ablation Completeness in Thermal Ablation of Liver Tumors. Front Oncol (2021) 11:623098. doi: 10.3389/fonc.2021.623098/full 33777768 PMC7988092

[33] Laimer G Schullian P Jaschke N Putzer D Eberle G Alzaga A. Minimal Ablative Margin (MAM) Assessment With Image Fusion: An Independent Predictor for Local Tumor Progression in Hepatocellular Carcinoma After Stereotactic Radiofrequency Ablation. Eur Radiol (2020) 30(5):2463–72. doi: 10.1007/s00330-019-06609-7 PMC7160081 32002642

[34] Wang X Sofocleous CT Erinjeri JP Petre EN Gonen M Do KG. Margin Size is an Independent Predictor of Local Tumor Progression After Ablation of Colon Cancer Liver Metastases. Cardiovasc Intervent Radiol (2013) 36(1):166–75. doi: 10.1007/s00270-012-0377-1 PMC4122121 22535243

[35] Hendriks P Noortman WA Baetens TR van Erkel AR van Rijswijk CSP van der Meer RW. Quantitative Volumetric Assessment of Ablative Margins in Hepatocellular Carcinoma: Predicting Local Tumor Progression Using Nonrigid Registration Software. J Oncol (2019) 2019:1–8. doi: 10.1155/2019/4049287 PMC6770329 31641353

[36] Anderson BM Lin Y Lin EY Cazoulat G Gupta S Jones AK. A Novel Use of Biomechanical Model Based Deformable Image Registration (DIR) for Assessing Colorectal Liver Metastases Ablation Outcomes. Med Phys (2021) p. 1–11 mp.15147. doi: 10.1002/mp.15147 34342018 PMC9380122

[37] Gillams A Goldberg N Ahmed M Bale R Breen D Callstrom M. Thermal Ablation of Colorectal Liver Metastases: A Position Paper by an International Panel of Ablation Experts, the Interventional Oncology Sans Frontières Meeting 2013. Eur Radiol (2015) 25(12):3438–54. doi: 10.1007/s00330-015-3779-z PMC4636513 25994193

