# Supplementary material for: 3D Quantitative Ablation Margins for Prediction of Ablation Site Recurrence After Stereotactic Image-Guided Microwave Ablation of Colorectal Liver Metastases: A Multicenter Study
Source: Front Oncol. 2021 Nov 15;11:757167. doi: 10.3389/fonc.2021.757167 (PMC8634106; doi:10.3389/fonc.2021.757167)
Supplement: Supplementary file 1 [file DataSheet_1.pdf]

3D Quantitative Ablation Margins for Prediction of Ablation Site Recurrence After Stereotactic Image-Guided Microwave Ablation of Colorectal Liver Metastasis: A Multicenter Study

Code of the statistical analysis

29 October 2021

Contents

Setup **2**

Read data **3**

Diagnose missing data 4

ROC Analysis **9**

Visualize the different ROC curves for each bin 10

Create new variable with best test 13

ROC analysis for MAM 14

Compare ROCs 15

Numerical results of margins **17**

Minimum Margin 17

Median margin 17

Maximum margin 18

Margin below 0 18

Groups without ASR 19

Multivariate analysis using GEE **20**

Baseline without margin (Model A) 20

Minimum Ablation Margin (Model B) 21

Best margin from ROC analysis (Model C) 22

Best cutoff < 5 mm margin (Model D) 23

Univariate analysis **25**

Setup

```r
library(haven)
library(geepack)
library(pROC)
library(tibble)
library(jstable)
library(ggpubr)
library(finalfit)
library(tidyr)
library(dplyr)
library(MissMech)
library(ggsci)
source("mcar.R")
```

Read data

```
data <- read_sav("MAVERRIC.sav")
nrow(data)
```

```
## [1] 168
```

```
data$Patient_ID <- as.factor(data$Patient_ID)
data$oneyear_ASR <- data$oneyear_ASR == 1
data$mut_KRAS <- data$mut_KRAS == 2
data$PR <- data$PR == 1
data$CEA_pre <- as.numeric(data$CEA_pre)
data$vessel <- data$vessel == 1
data$Diameter <- as.numeric(data$Diameter)
data$Margin_available <- data$Margin_available == 1
data$Chemo <- data$Chemo == 1
head(data)
```

```
## # A tibble: 6 x 41
##   Patient_ID_simple REDCAP_ID Patient_ID tumor tumor_id   Age PR
##               <dbl> <chr>     <fct>      <dbl> <chr>    <dbl> <lgl>
## 1                 1 6-1       B01            1 T001        74 FALSE
## 2                 1 6-1       B01            2 T002        74 FALSE
## 3                 2 6-2       B02            1 T003        69 TRUE
## 4                 3 6-3       B03            1 T004        65 FALSE
## 5                 4 6-4       B04            1 T005        70 TRUE
## 6                 4 6-4       B04            2 T006        70 TRUE
## # ... with 34 more variables: prim_location_group <dbl+lbl>,
## #   PA_veninvasion <dbl+lbl>, Nstage_cat <dbl>, Chemo <lgl>,
## #   Number_abl_lesions <dbl>, vessel <lgl>, CEA_pre <dbl>, mut_KRAS <lgl>,
## #   lat_error <dbl>, oneyear_ASR <lgl>, Diameter <dbl>, Margin_available <lgl>,
## #   margin_min <dbl>, margin_q25 <dbl>, margin_median <dbl>, margin_q75 <dbl>,
## #   margin_max <dbl>, belown5 <dbl>, belown4 <dbl>, belown3 <dbl>,
## #   belown2 <dbl>, belown1 <dbl>, below0 <dbl>, below1 <dbl>, below2 <dbl>, ...
```

Diagnose missing data

```
data.diagnose <- data.frame(PatientId = data$Patient_ID, TumorId = data$tumor,
                            mut_KRAS = data$mut_KRAS, PR = data$PR, vessel = data$vessel,
                            Diameter = data$Diameter, CEA_pre = data$CEA_pre,
                            Chemo = data$Chemo, oneyear_ASR = data$oneyear_ASR,
                            margin = data$margin_min)

ff_glimpse(data.diagnose)
```

```
## $Continuous
##                                 label var_type   n missing_n missing_percent
## TumorId                       TumorId    <dbl> 168         0             0.0
## Diameter                     Diameter    <dbl> 168         0             0.0
## CEA_pre                       CEA_pre    <dbl> 161         7             4.2
## margin   Minimal ablation margin (mm)    <dbl>  65       103            61.3
##          mean   sd  min quartile_25 median quartile_75   max
## TumorId   1.6  0.9  1.0         1.0    1.0         2.0   5.0
## Diameter 13.8  8.8  0.0         8.0   13.0        20.0  45.0
## CEA_pre  11.6 30.9  0.5         2.1    3.6         8.4 319.0
## margin    0.2  2.7 -6.8        -1.8    0.0         2.1   8.4
##
## $Categorical
##                   label var_type   n missing_n missing_percent levels_n levels
## PatientId     PatientId    <fct> 168         0             0.0       98      -
## mut_KRAS       mut_KRAS    <lgl> 156        12             7.1        2      -
## PR                   PR    <lgl> 168         0             0.0        2      -
## vessel           vessel    <lgl> 168         0             0.0        2      -
## Chemo             Chemo    <lgl> 168         0             0.0        2      -
## oneyear_ASR oneyear_ASR    <lgl> 168         0             0.0        2      -
##             levels_count levels_percent
## PatientId              -              -
## mut_KRAS               -              -
## PR                     -              -
## vessel                 -              -
## Chemo                  -              -
## oneyear_ASR            -              -
```

```
missing_plot(data.diagnose)
```

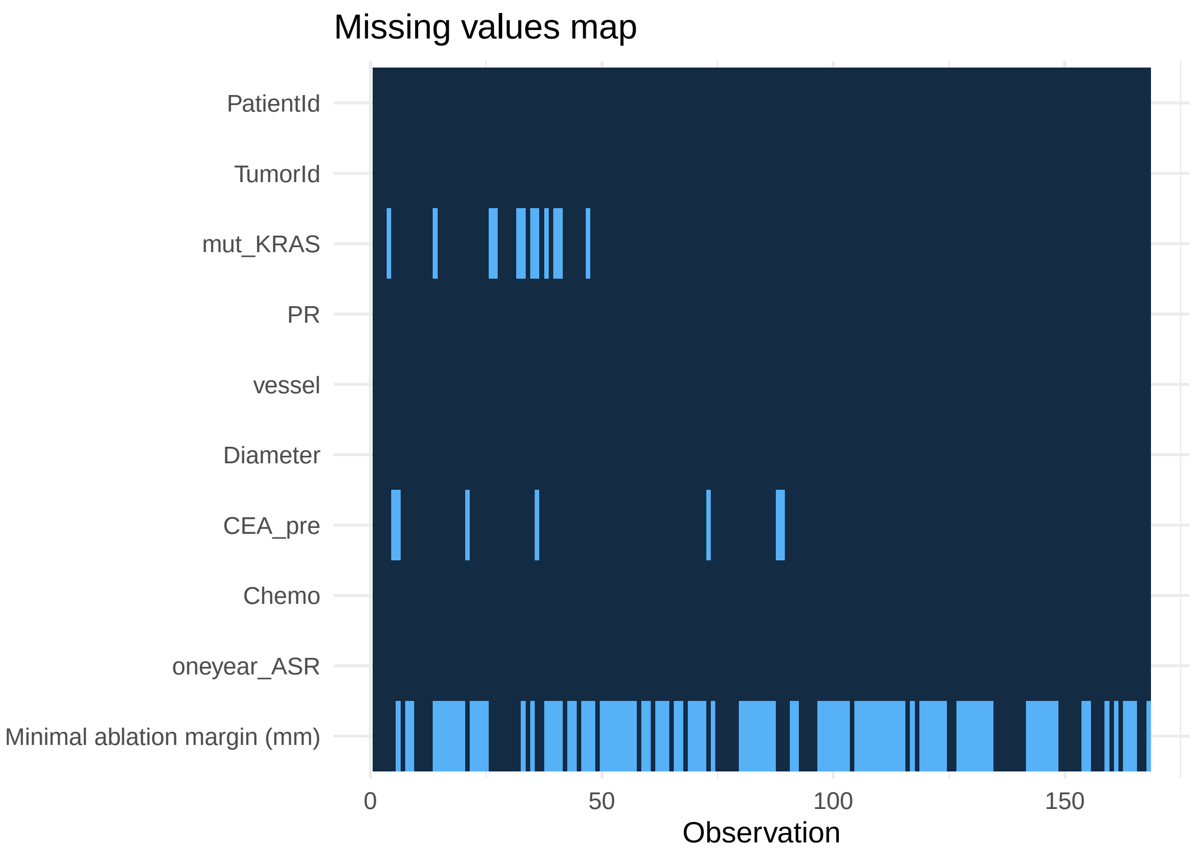


```r
dependent <- "oneyear_ASR"
explanatory <- c("mut_KRAS", "CEA_pre", "margin")

missing_pattern(.data = data.diagnose, dependent = dependent, explanatory = explanatory)
```

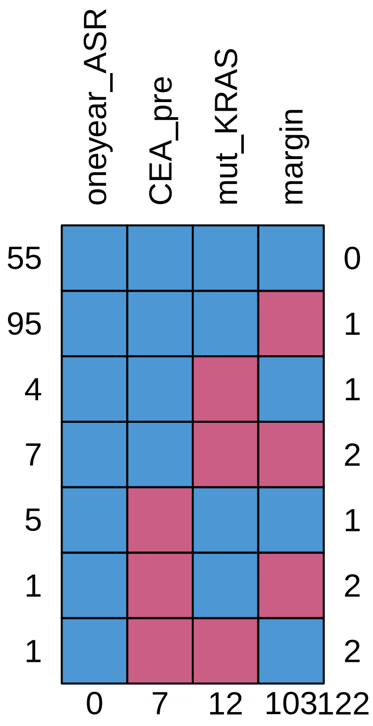


```
##    oneyear_ASR CEA_pre mut_KRAS margin
## 55           1       1        1      1   0
## 95           1       1        1      0   1
## 4            1       1        0      1   1
## 7            1       1        0      0   2
## 5            1       0        1      1   1
## 1            1       0        1      0   2
## 1            1       0        0      1   2
##              0       7       12    103 122
```

```
summary_factorlist(.data = data.diagnose, dependent = dependent, explanatory = explanatory,
  na_include=TRUE, p=TRUE)
```

```
##                          label     levels       FALSE        TRUE     p
##                       mut_KRAS      FALSE   65 (50.8)   19 (67.9) 0.152
##                                      TRUE   63 (49.2)    9 (32.1)
##                        CEA_pre Mean (SD) 10.1 (20.4) 19.0 (61.7) 0.181
##  Minimal ablation margin (mm) Mean (SD)   0.7 (2.6)  -2.2 (2.0) 0.002
```

```
check_variables <- c("oneyear_ASR", "Diameter", "mut_KRAS", "CEA_pre", "PR",
                     "vessel", "Chemo")

data.diagnose %>%
  missing_compare("margin", check_variables, na_include = TRUE) %>%
    knitr::kable(row.names=FALSE, align = c("l", "l", "r", "r", "r"))
```

Missing data analysis: Minimal ablation margin (mm)		Not missing	Missing	p
oneyear_ASR	FALSE	55 (39.3)	85 (60.7)	0.887
	TRUE	10 (35.7)	18 (64.3)	
Diameter	Mean (SD)	15.0 (7.6)	13.0 (9.4)	0.158
mut_KRAS	FALSE	29 (34.5)	55 (65.5)	0.354
	TRUE	31 (43.1)	41 (56.9)	
CEA_pre	Mean (SD)	5.5 (6.2)	15.1 (38.1)	0.058
PR	FALSE	57 (40.4)	84 (59.6)	0.401
	TRUE	8 (29.6)	19 (70.4)	
vessel	FALSE	49 (35.5)	89 (64.5)	0.107
	TRUE	16 (53.3)	14 (46.7)	
Chemo	FALSE	43 (43.9)	55 (56.1)	0.141
	TRUE	22 (31.4)	48 (68.6)	

```
data.diagnose %>%
  missing_compare("mut_KRAS", "oneyear_ASR", na_include = TRUE) %>%
    knitr::kable(row.names=FALSE, align = c("l", "l", "r", "r", "r"))
```

```
## Warning in chisq.test(oneyear_ASR, mut_KRAS): Chi-squared approximation may be
## incorrect
```

Missing data analysis: mut_KRAS		Not missing	Missing	p
oneyear_ASR	FALSE	128 (91.4)	12 (8.6)	0.228
	TRUE	28 (100.0)		

```
data.diagnose %>%
  missing_compare("CEA_pre", "oneyear_ASR", na_include = TRUE) %>%
    knitr::kable(row.names=FALSE, align = c("l", "l", "r", "r", "r"))
```

```
## Warning in chisq.test(oneyear_ASR, CEA_pre): Chi-squared approximation may be
## incorrect
```

Missing data analysis: CEA_pre		Not missing	Missing	p
oneyear_ASR	FALSE	135 (96.4)	5 (3.6)	0.730
	TRUE	26 (92.9)	2 (7.1)	

```
data.diagnose.mat <- sapply(data.diagnose, as.numeric)
mcarn <- TestMCARNormality(data = data.diagnose.mat)
summary(mcarn)
```

```
##
## Number of imputation:  1
##
## Number of Patterns:  3
##
## Total number of cases used in the analysis:  157
##
```

```
##  Pattern(s) used:
##           PatientId   TumorId   mut_KRAS   PR   vessel   Diameter   CEA_pre
## group.1          1         1          1    1        1          1         1
## group.2          1         1          1    1        1          1         1
## group.3          1         1         NA    1        1          1         1
##           Chemo   oneyear_ASR   margin   Number of cases
## group.1       1             1        1                55
## group.2       1             1       NA                95
## group.3       1             1       NA                 7
##
##
##     Test of normality and Homoscedasticity:
##   -------------------------------------------
##
## Hawkins Test:
##
##     P-value for the Hawkins test of normality and homoscedasticity:  0.1052516
##
## Non-Parametric Test:
##
##     P-value for the non-parametric test of homoscedasticity:  0.6060996
```

Interpretation: Missing margins seem to be equally distributed among lesions with ASR and tumors wihtout ASR.

ROC Analysis

From the QAM metrics we have multiple bins in the histogram. We use the ROC analysis to identify the best bin and its respective threshold.

```
ROC_threshold <- function(truth, prediction) {
  ROC <- roc(truth, prediction)
  coords(ROC, x = "best", best.method = "youden")
  #ROC_table <- cbind(ROC$thresholds, ROC$specificities, ROC$sensitivities, quiet = TRUE)
  #ROC_table[which.max(ROC_table[, 2] + ROC_table[, 3]), ]
}

asr.roc.tb <- data.frame(column_name = c(paste0("below", seq(0, 10)), "mam"),
                         below = rep(NA, 12),
                         best_threshold = rep(NA, 12),
                         auc = rep(NA, 12),
                         ci_lower = rep(NA, 12),
                         ci_upper = rep(NA, 12),
                         specificity = rep(NA, 12),
                         sensitivity = rep(NA, 12),
                         row.names = "column_name")

for (i in seq(0, 10)){
  col_name <- paste0("below",i)
  asr.roc <- roc(data$oneyear_ASR, deframe(data[,col_name]), quiet = TRUE, ci = TRUE)
  asr.roc.best <- ROC_threshold(data$oneyear_ASR, deframe(data[,col_name]))
  asr.roc.tb[col_name, ] <- cbind(i, asr.roc.best[1], asr.roc$auc, asr.roc$ci[1],
                                  asr.roc$ci[3], asr.roc.best[2], asr.roc.best[3])
}

best_model <- asr.roc.tb[which.max(asr.roc.tb$auc),]
best_model_name <- rownames(best_model)

col_name <- "mam"
asr.roc <- roc(data$oneyear_ASR, data$margin_min, quiet = TRUE, ci = TRUE)
asr.roc.best <- ROC_threshold(data$oneyear_ASR, data$margin_min)
asr.roc.tb[col_name, ] <- cbind(-1, asr.roc.best[1], asr.roc$auc, asr.roc$ci[1],
                                asr.roc$ci[3], asr.roc.best[2], asr.roc.best[3])
```

```
print(asr.roc.tb)
```

```
##         below best_threshold       auc  ci_lower  ci_upper specificity
## below0      0     0.00677705 0.7672727 0.6039320 0.9306135   0.7272727
## below1      1     0.22994779 0.7681818 0.5990303 0.9373333   0.8545455
## below2      2     0.28732107 0.7600000 0.5983379 0.9216621   0.8000000
## below3      3     0.43677935 0.7672727 0.6152208 0.9193246   0.7818182
## below4      4     0.31956262 0.7327273 0.5784110 0.8870436   0.4363636
## below5      5     0.44688372 0.6927273 0.5311755 0.8542791   0.3454545
## below6      6     0.84043879 0.6354545 0.4637849 0.8071242   0.6727273
## below7      7     0.80441083 0.5872727 0.4152849 0.7592606   0.3636364
## below8      8     0.90426849 0.5690909 0.4146223 0.7235595   0.4181818
## below9      9     0.98390753 0.6236364 0.4580009 0.7892718   0.4181818
## below10    10     0.99919614 0.6372727 0.4997966 0.7747489   0.4000000
```

```
## mam        -1    -1.13465810 0.8236364 0.6796821 0.9675906   0.8000000
##          sensitivity
## below0           0.8
## below1           0.7
## below2           0.7
## below3           0.7
## below4           1.0
## below5           1.0
## below6           0.6
## below7           0.9
## below8           0.9
## below9           0.9
## below10          0.9
## mam              0.8
```

Visualize the different ROC curves for each bin

```
color_1mm <- pal_npg("nrc")(1)

roc.list <- roc(data$oneyear_ASR ~ below0  + below2 + below3 + below4 + below5 + below6 +
                  below7 + below8 + below9 + below10 + below1,
                data = data, quiet = TRUE, ci = TRUE)

g.list <- ggroc(roc.list, legacy.axes = TRUE) +
  geom_segment(aes(x = 0, y = 0, xend = 1,yend = 1), alpha = 0.5,
               color = "black", linetype = 2) +
  coord_fixed(ratio = 0.9) +
  scale_color_manual(name = "Model", labels = c("<1mm", "<5mm", "MAM"),
                     values = c("darkgray", "darkgray", "darkgray", "darkgray",
                                "darkgray", "darkgray", "darkgray", "darkgray",
                                "darkgray", "darkgray", color_1mm)) +
  xlab("False Positive Rate") + ylab("True Positive Rate") +
  theme_pubr() +
  theme(legend.position="none")
g.list + facet_wrap(.~name, nrow = 4)
```

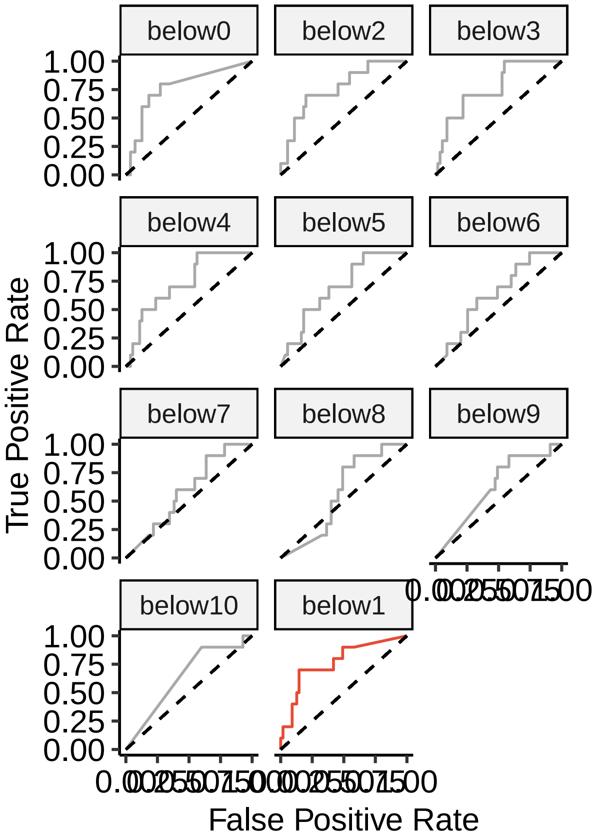
below0
below2
below3
below4
below5
below6
below7
below8
below9
below10
below1
True Positive Rate
False Positive Rate
1.00
0.75
0.50
0.25
0.00

```
g.list
```

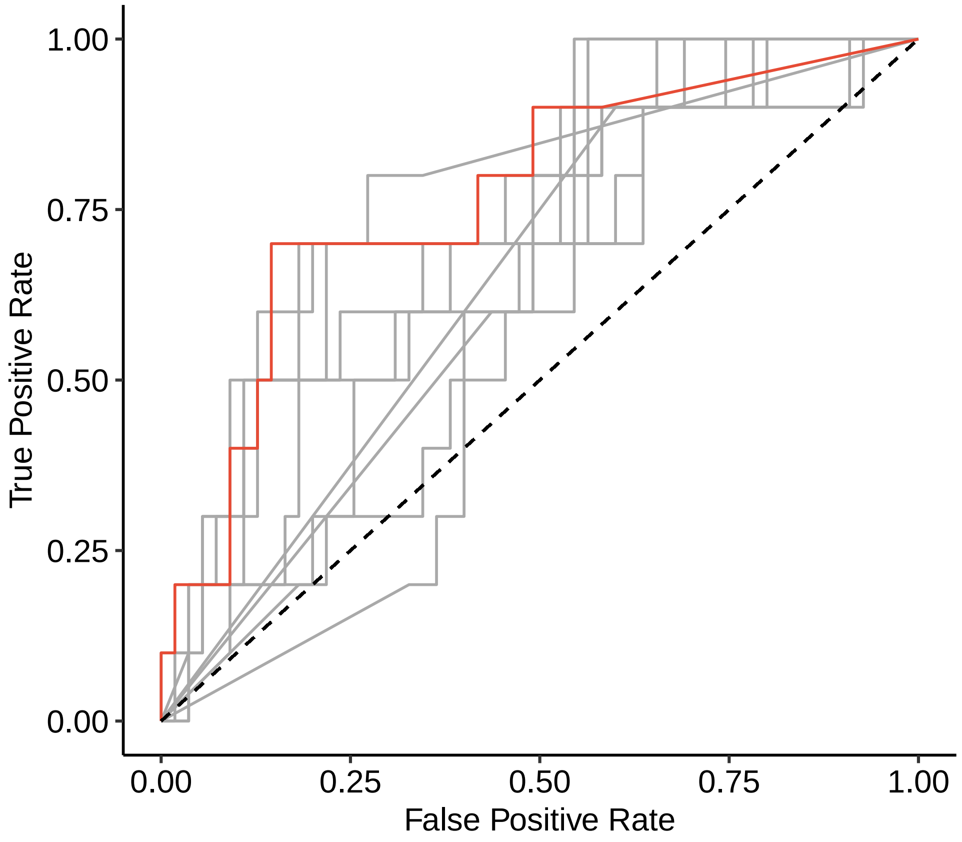


```
ggsave("figures/Figure_4a.png", g.list, width = 184, height = 184, units = "mm", dpi = 300)
ggsave("figures/Figure_4a.pdf", g.list, width = 184, height = 184, units = "mm")
```

##Select best bin and threshold

```
asr.roc.best <- roc(data$oneyear_ASR, deframe(data[,best_model_name]),
                    quiet = TRUE, ci = TRUE)

ggroc(asr.roc.best, size = 1) +
  geom_segment(aes(x = 0, y = 1, xend = 1,yend = 0), alpha = 0.5, color = "black",
               linetype = 2) +
  scale_y_continuous(limits = c(0, 1), expand = c(0, 0)) +
  scale_x_reverse(expand = c(0, 0)) +
  annotate(geom = "text", x = 0.25, y = 0.5,
           label = sprintf("AUC: %.3f (%.3f, %.3f)", asr.roc.best$auc,
                           asr.roc.best$ci[1],asr.roc.best$ci[3])) +
  labs(title = "ROC curve for % of margin < 1 mm",
       x = "1 - Specificity", y = "Sensitivity") +
  theme_pubr() +
  theme(plot.title = element_text(hjust = 0.5))
```

```
## Scale for 'x' is already present. Adding another scale for 'x', which will
## replace the existing scale.
```

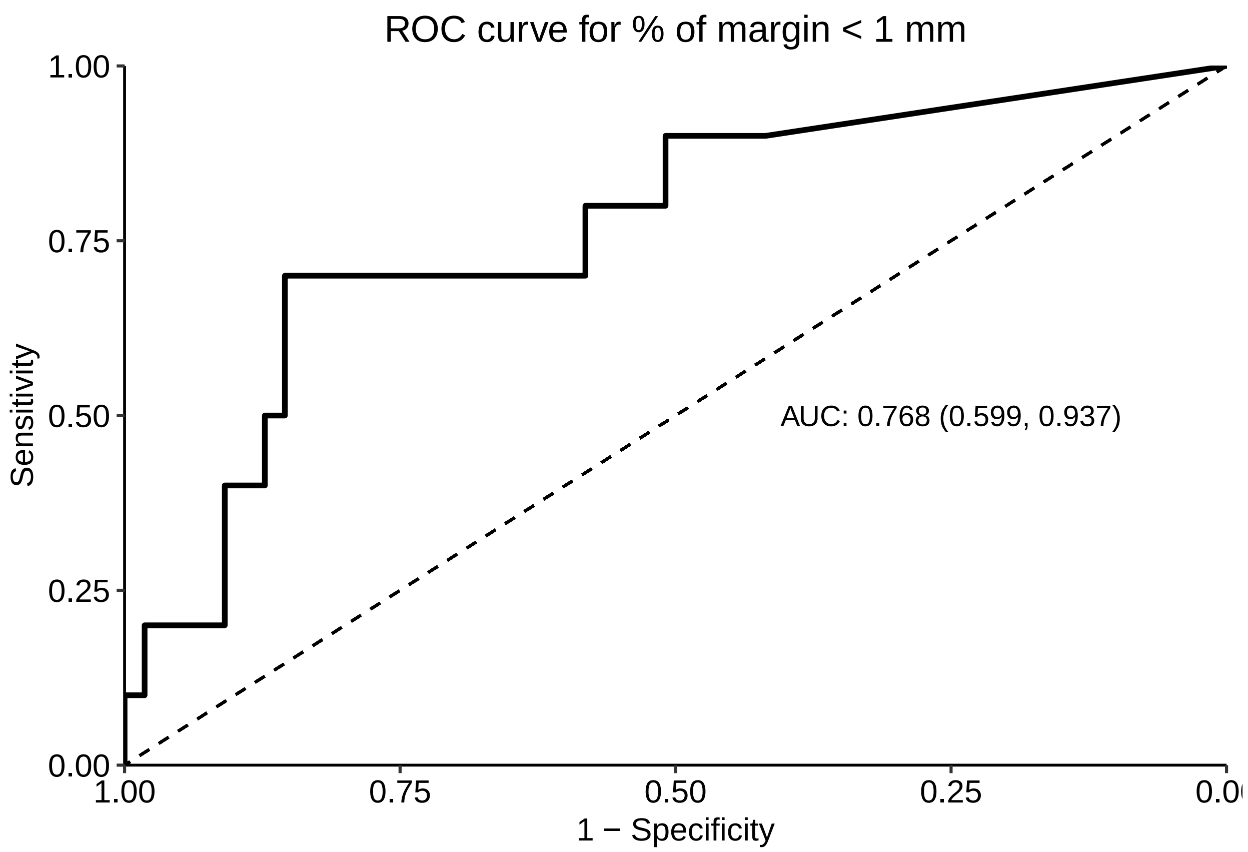


```
sprintf("Best model is %s with threshold %.2f and AUC %.3f",
        best_model_name, best_model$best_threshold, best_model$auc)
```

```
## [1] "Best model is below1 with threshold 0.23 and AUC 0.768"
```

Create new variable with best test

Using the best bin and threshold from above we create a new variable margin_best.

```
data$margin_best <- data[, best_model_name] > best_model$best_threshold
summary(data$margin_best)
```

```
##    below1
##  Mode :logical
##  FALSE:50
##  TRUE :15
##  NA's :103
```

ROC analysis for MAM

```r
asr.roc.mam <- roc(data$oneyear_ASR, data$margin_min, quiet = TRUE, ci = TRUE)
ggroc(asr.roc.mam, size = 1) +
  geom_segment(aes(x = 0, y = 1, xend = 1,yend = 0), alpha = 0.5, color = "black",
               linetype = 2) +
  scale_y_continuous(limits = c(0, 1), expand = c(0, 0)) +
  scale_x_reverse(expand = c(0, 0)) +
  annotate(geom = "text", x = 0.25, y = 0.5,
           label = sprintf("AUC: %.3f (%.3f, %.3f)",
                           asr.roc.mam$auc, asr.roc.mam$ci[1], asr.roc.mam$ci[3])) +
  labs(title = "ROC curve for MAM", x = "1 - Specificity", y = "Sensitivity") +
  theme_pubr() +
  theme(plot.title = element_text(hjust = 0.5))
```

```
## Scale for 'x' is already present. Adding another scale for 'x', which will
## replace the existing scale.
```

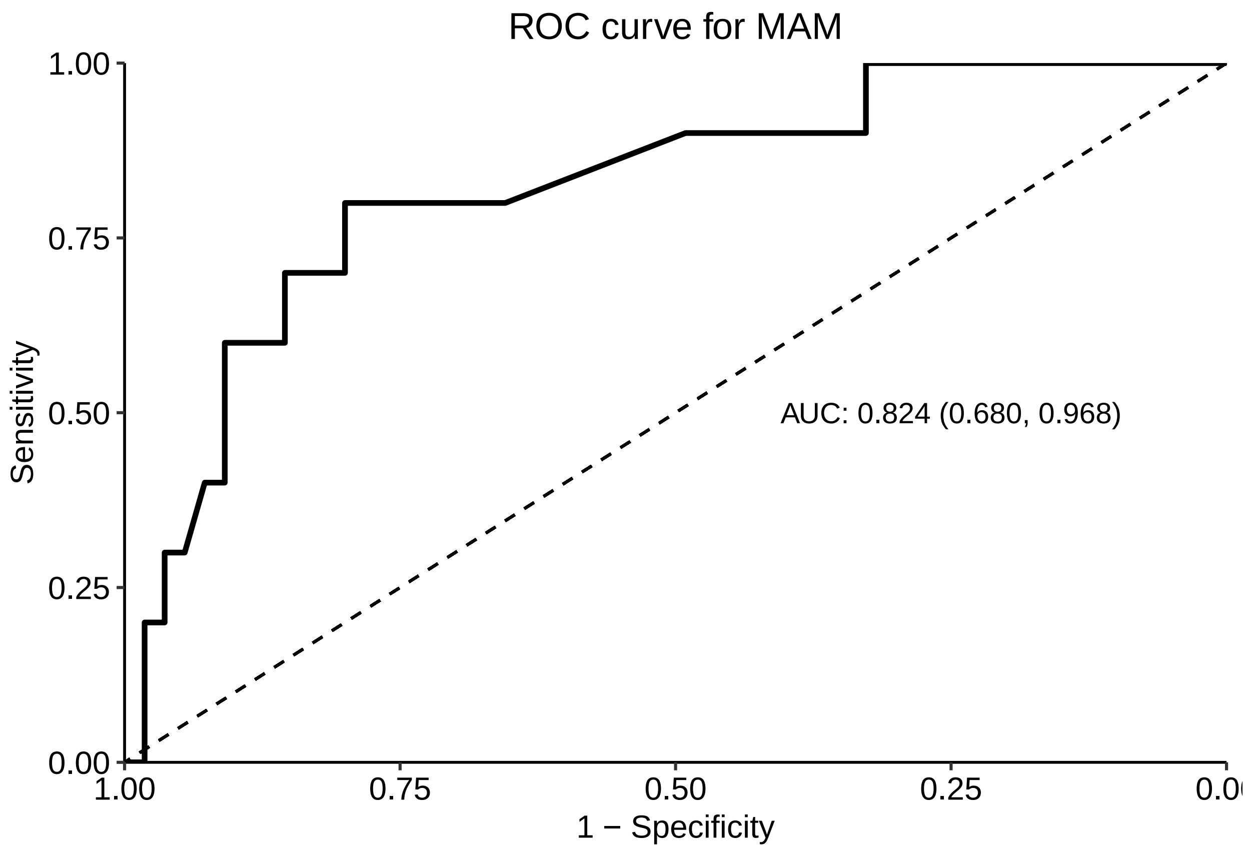


```r
sprintf("MAM model with threshold %.2f and AUC %.3f",
        best_model$best_threshold, best_model$auc)
```

```
## [1] "MAM model with threshold 0.23 and AUC 0.768"
```

Compare ROCs

Compare the ROC curves for minimum ablation margin and "our" optimal threshold based on the histogram

```
# roc.test(asr.roc.best, asr.roc.mam)

best_thresholds <- as.data.frame(asr.roc.tb)
best_thresholds <- best_thresholds[best_thresholds$below == 1 |
                                   best_thresholds$below == 5 |
                                   best_thresholds$below == -1,]

best_thresholds
```

```
##        below best_threshold       auc  ci_lower  ci_upper specificity
## below1     1      0.2299478 0.7681818 0.5990303 0.9373333   0.8545455
## below5     5      0.4468837 0.6927273 0.5311755 0.8542791   0.3454545
## mam       -1     -1.1346581 0.8236364 0.6796821 0.9675906   0.8000000
##        sensitivity
## below1         0.7
## below5         1.0
## mam            0.8
```

```
roc.list <- roc(data$oneyear_ASR ~ below1 + below5 + margin_min,
                data = data, quiet = TRUE, ci = TRUE)
roc.list
```

```
## $below1
##
## Call:
## roc.formula(formula = data$oneyear_ASR ~ below1, data = data,     quiet = TRUE, ci = TRUE)
##
## Data: below1 in 55 controls (data$oneyear_ASR FALSE) < 10 cases (data$oneyear_ASR TRUE).
## Area under the curve: 0.7682
## 95% CI: 0.599-0.9373 (DeLong)
##
## $below5
##
## Call:
## roc.formula(formula = data$oneyear_ASR ~ below5, data = data,     quiet = TRUE, ci = TRUE)
##
## Data: below5 in 55 controls (data$oneyear_ASR FALSE) < 10 cases (data$oneyear_ASR TRUE).
## Area under the curve: 0.6927
## 95% CI: 0.5312-0.8543 (DeLong)
##
## $margin_min
##
## Call:
## roc.formula(formula = data$oneyear_ASR ~ margin_min, data = data,     quiet = TRUE, ci = TRUE)
##
## Data: margin_min in 55 controls (data$oneyear_ASR FALSE) > 10 cases (data$oneyear_ASR TRUE).
## Area under the curve: 0.8236
## 95% CI: 0.6797-0.9676 (DeLong)
```

```
g.list <- ggroc(roc.list, size = 1, legacy.axes = TRUE) +
  geom_segment(aes(x = 0, y = 0, xend = 1,yend = 1), alpha = 0.5, color = "black",
               linetype = 2) +
  annotate("point", x = 1-best_thresholds$specificity, y = best_thresholds$sensitivity,
           shape = 8, size = 2) +
  coord_fixed(ratio = 0.9) +
  scale_color_discrete(name = "Model", labels = c("<1mm", "<5mm", "MAM")) +
  xlab("False Positive Rate") + ylab("True Positive Rate") +
  theme_pubr()
g.list
```

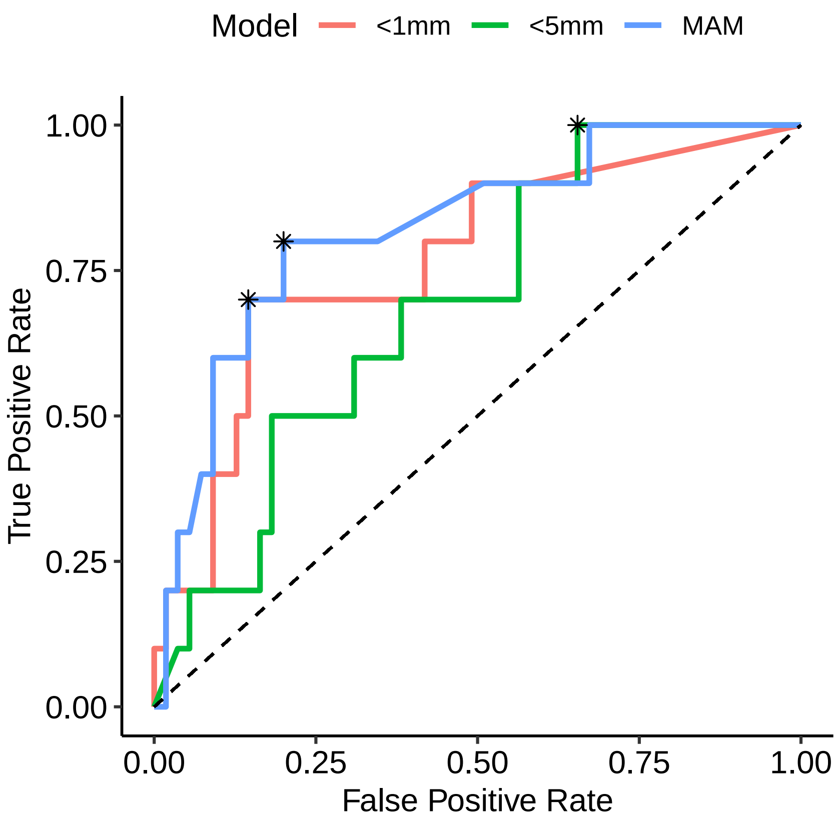


```
ggsave("figures/Figure_4b.png", g.list, width = 184, height = 184, units = "mm", dpi = 300)
ggsave("figures/Figure_4b.pdf", g.list, width = 184, height = 184, units = "mm")
```

Interpretation: Using the minimum ablation margin seems to be a more accurate predictor but not significantly different.

Numerical results of margins

Minimum Margin

```
data %>%
  filter(Margin_available) %>%
  group_by(oneyear_ASR) %>%
    summarize(median = median(margin_min),
    q25 = quantile(margin_min, 0.25),
    q75 = quantile(margin_min, 0.75))
```

```
## # A tibble: 2 x 4
##   oneyear_ASR median    q25   q75
##   <lgl>        <dbl>  <dbl> <dbl>
## 1 FALSE         0    -0.860  2.41
## 2 TRUE         -2.42 -3.22  -1.41
```

```
wilcox.test(margin_min ~ oneyear_ASR, data = data)
```

```
##
##  Wilcoxon rank sum test with continuity correction
##
## data:  margin_min by oneyear_ASR
## W = 453, p-value = 0.001224
## alternative hypothesis: true location shift is not equal to 0
```

Median margin

```
data %>%
  filter(Margin_available) %>%
  group_by(oneyear_ASR) %>%
    summarize(median = median(margin_median),
    q25 = quantile(margin_median, 0.25),
    q75 = quantile(margin_median, 0.75))
```

```
## # A tibble: 2 x 4
##   oneyear_ASR median   q25   q75
##   <lgl>        <dbl> <dbl> <dbl>
## 1 FALSE         4.60  3.53  5.74
## 2 TRUE          2.72  1.78  4.51
```

```
wilcox.test(margin_median ~ oneyear_ASR, data = data)
```

```
##
##  Wilcoxon rank sum test with continuity correction
##
## data:  margin_median by oneyear_ASR
## W = 402, p-value = 0.02144
## alternative hypothesis: true location shift is not equal to 0
```

Maximum margin

```
data %>%
  filter(Margin_available) %>%
  group_by(oneyear_ASR) %>%
    summarize(median = median(margin_max),
    q25 = quantile(margin_max, 0.25),
    q75 = quantile(margin_max, 0.75))
```

```
## # A tibble: 2 x 4
##   oneyear_ASR median   q25   q75
##   <lgl>        <dbl> <dbl> <dbl>
## 1 FALSE         9.33  7.58 10.6
## 2 TRUE          8.84  8.42  9.55
```

```
wilcox.test(margin_max ~ oneyear_ASR, data = data)
```

```
##
##  Wilcoxon rank sum test with continuity correction
##
## data:  margin_max by oneyear_ASR
## W = 313, p-value = 0.4953
## alternative hypothesis: true location shift is not equal to 0
```

Margin below 0

```
data %>%
  filter(Margin_available) %>%
  group_by(oneyear_ASR) %>%
    summarize(median = median(below0),
    q25 = quantile(below0, 0.25),
    q75 = quantile(below0, 0.75))
```

```
## # A tibble: 2 x 4
##   oneyear_ASR median    q25     q75
##   <lgl>        <dbl>  <dbl>   <dbl>
## 1 FALSE        0      0      0.00903
## 2 TRUE         0.115  0.0191 0.189
```

```
wilcox.test(below0 ~ oneyear_ASR, data = data)
```

```
##
##  Wilcoxon rank sum test with continuity correction
##
## data:  below0 by oneyear_ASR
## W = 128, p-value = 0.002904
## alternative hypothesis: true location shift is not equal to 0
```

Groups without ASR

```
table(data$below2 == 0, data$oneyear_ASR)
```

```
##
##         FALSE TRUE
##   FALSE    38   10
##   TRUE     17    0
```

```
table(data$below3 <= 0.1, data$oneyear_ASR)
```

```
##
##         FALSE TRUE
##   FALSE    33   10
##   TRUE     22    0
```

Interpretation: No ASR occurred in tumors with 100% 3D-QAM of >=2mm and 90% 3D-QAM of >=3mm.

Multivariate analysis using GEE

General Remarks

- Correlation structure **independence** is used because cluster sizes are small (each patient is a cluster).
- Different models were built with and without margin as explanatory variable due to the large proportion of missing margin data. The reason for missing data is unrelated to other factors and depended on tumor visibility, registration accuracy and ability to acquire additional contrast CT scans.
- GEE model with binomial outcome (ASR yes/no)

Baseline without margin (Model A)

First we use a baseline model without the margin, because margin has lots of missing data.

```
complete_subset <- complete.cases(data[, c("oneyear_ASR", "Chemo", "PR", "vessel",
                                           "Diameter", "CEA_pre", "mut_KRAS")])
data.asr.baseline <- data[complete_subset, ]
sprintf("N = %d/%d", sum(complete_subset), length(complete_subset))
```

```
## [1] "N = 150/168"
```

```
asr.gee.baseline <- geeglm(oneyear_ASR ~ Chemo + PR + vessel + mut_KRAS +
                           CEA_pre + Diameter ,
               id = Patient_ID, corstr = "independence",
               data = data.asr.baseline, family = "binomial")

geeglm.display(asr.gee.baseline)
```

```
## $caption
## [1] "GEE(binomial) predicting oneyear_ASR by Chemo, PR, vessel, mut_KRAS, CEA_pre, Diameter - Group I
##
## $table
##              crude OR(95%CI)    crude P value adj. OR(95%CI)     adj. P value
## ChemoTRUE    "0.52 (0.18,1.56)" "0.244"       "1.22 (0.33,4.54)" "0.766"
## PRTRUE       "0.24 (0.03,2)"    "0.185"       "0.12 (0.01,1.29)" "0.08"
## vesselTRUE   "1.92 (0.68,5.42)" "0.22"        "1.89 (0.52,6.85)" "0.334"
## mut_KRASTRUE "0.44 (0.16,1.25)" "0.124"       "0.29 (0.09,0.97)" "0.044"
## CEA_pre      "1.01 (1,1.02)"    "0.039"       "1.01 (1,1.02)"    "0.192"
## Diameter     "1.11 (1.05,1.18)" "< 0.001"     "1.11 (1.05,1.18)" "< 0.001"
##
## $metric
##                                    crude OR(95%CI) crude P value adj. OR(95%CI)
##                                    NA              NA            NA
## Estimated correlation parameters NA              NA            NA
## No. of clusters                    "85"            NA            NA
## No. of observations                "150"           NA            NA
##                                    adj. P value
##                                    NA
## Estimated correlation parameters NA
## No. of clusters                    NA
## No. of observations                NA
```

Interpretation: According to the baseline model, increased tumor size significantly increases the odds of ASR and KRAS mutant tumors significantly decrease odds of ASR. Previous resection tends to decrease odds of ASR.

Interpretation: If ~25% or more of tumor surface is covered with less than 1 mm margin, the odds of ASR is 21.67 times higher. Compared to the margin, tumor size has a much smaller effect on the odds. **Interpretation:** Compared to the baseline model, previous resection and KRAS mutation have less impact on the odds of ASR. CEA has no influence compared to the baseline model. However, this could be due to the difference in distribution in the subset with missing margins and the subset without missin margins. Also, the two models are significantly different.

Minimum Ablation Margin (Model B)

For comparison with other studies who use the minimum margin (MAM), create a model with this parameter instead of margin_best.

```
complete_subset <- complete.cases(data[, c("oneyear_ASR", "Diameter","PR", "vessel",
                                           "CEA_pre", "mut_KRAS",  "margin_min")])
data.asr.mam <- data[complete_subset, ]


asr.gee.mam <- geeglm(oneyear_ASR ~ Chemo + PR + vessel + mut_KRAS + CEA_pre +
                        Diameter + margin_min,
                  id = Patient_ID, corstr = "independence",
                  data = data.asr.mam, family = "binomial")

asr.gee.mam.baseline <- geeglm(oneyear_ASR ~ Chemo + PR + vessel + mut_KRAS +
                                 CEA_pre + Diameter,
               id = Patient_ID, corstr = "independence",
               data = data.asr.mam, family = "binomial")

sprintf("N = %d/%d", sum(complete_subset), length(complete_subset))
```

```
## [1] "N = 55/168"
```

```
geeglm.display(asr.gee.mam)
```

```
## $caption
## [1] "GEE(binomial) predicting oneyear_ASR by Chemo, PR, vessel, mut_KRAS, CEA_pre, Diameter, margin_
##
## $table
##                crude OR(95%CI)     crude P value adj. OR(95%CI)      adj. P value
## ChemoTRUE      "1.65 (0.24,11.61)" "0.613"       "4.14 (0.52,33.18)" "0.181"
## PRTRUE         "1.31 (0.11,16.11)" "0.832"       "0.3 (0,461.69)"    "0.748"
## vesselTRUE     "2.88 (0.77,10.84)" "0.118"       "1.94 (0.23,16.53)" "0.544"
## mut_KRASTRUE   "0.67 (0.11,4.06)"  "0.665"       "0.47 (0.07,3.4)"   "0.455"
## CEA_pre        "0.99 (0.92,1.08)"  "0.902"       "1.02 (0.86,1.2)"   "0.858"
## Diameter       "1.14 (1.03,1.26)"  "0.01"        "1.1 (0.98,1.25)"   "0.116"
## margin_min     "0.5 (0.26,0.96)"   "0.038"       "0.52 (0.29,0.95)"  "0.034"
##
## $metric
##                                    crude OR(95%CI) crude P value adj. OR(95%CI)
```

```
##                                  NA               NA             NA
## Estimated correlation parameters NA               NA             NA
## No. of clusters                  "39"             NA             NA
## No. of observations              "55"             NA             NA
##                                  adj. P value
##                                  NA
## Estimated correlation parameters NA
## No. of clusters                  NA
## No. of observations              NA
```

```
anova(asr.gee.mam, asr.gee.mam.baseline)
```

```
## Analysis of 'Wald statistic' Table
##
## Model 1 oneyear_ASR ~ Chemo + PR + vessel + mut_KRAS + CEA_pre + Diameter + margin_min
## Model 2 oneyear_ASR ~ Chemo + PR + vessel + mut_KRAS + CEA_pre + Diameter
##   Df      X2 P(>|Chi|)
## 1  1 4.4724   0.03445 *
## ---
## Signif. codes:  0 '***' 0.001 '**' 0.01 '*' 0.05 '.' 0.1 ' ' 1
```

Interpretation: Similar to Bale et al. MAM significantly decreases the odds by 48% per mm increase in MAM. Bale et al. had 30%. In this case no other factor is significant. Also the model with only MAM is not significantly different from the model with all parameters.

Best margin from ROC analysis (Model C)

```
complete_subset <- complete.cases(data[, c("oneyear_ASR", "PR", "vessel", "Diameter",
                                           "CEA_pre", "mut_KRAS", "margin_best")])
data.asr.gee.margin <- data[complete_subset,]

asr.gee.margin <- geeglm(oneyear_ASR ~ Chemo + PR + vessel + mut_KRAS + CEA_pre +
                           Diameter + margin_best,
               id = Patient_ID, corstr = "independence",
               data = data.asr.gee.margin, family = "binomial")

asr.gee.baseline.margin <- geeglm(oneyear_ASR ~ Chemo + PR + vessel + mut_KRAS +
                                    CEA_pre + Diameter,
               id = Patient_ID, corstr = "independence",
               data = data.asr.gee.margin, family = "binomial")

sprintf("N = %d/%d", sum(complete_subset), length(complete_subset))
geeglm.display(asr.gee.margin)

anova(asr.gee.baseline.margin, asr.gee.margin)
```

```
## [1] "N = 55/168"
## $caption
## [1] "GEE(binomial) predicting oneyear_ASR by Chemo, PR, vessel, mut_KRAS, CEA_pre, Diameter, margin_
##
## $table
```

```
##                 crude OR(95%CI)      crude P value adj. OR(95%CI)
## ChemoTRUE       "1.65 (0.24,11.61)"  "0.613"       "1.97 (0.35,11.04)"
## PRTRUE          "1.31 (0.11,16.11)"  "0.832"       "0.79 (0.01,74.51)"
## vesselTRUE      "2.88 (0.77,10.84)"  "0.118"       "1.49 (0.15,14.45)"
## mut_KRASTRUE    "0.67 (0.11,4.06)"   "0.665"       "0.62 (0.08,4.61)"
## CEA_pre         "0.99 (0.92,1.08)"   "0.902"       "1.01 (0.87,1.17)"
## Diameter        "1.14 (1.03,1.26)"   "0.01"        "1.13 (1.01,1.27)"
## margin_bestTRUE "28.7 (4.13,199.44)" "< 0.001"     "21.67 (2.84,165.21)"
##                 adj. P value
## ChemoTRUE       "0.439"
## PRTRUE          "0.921"
## vesselTRUE      "0.73"
## mut_KRASTRUE    "0.641"
## CEA_pre         "0.886"
## Diameter        "0.035"
## margin_bestTRUE "0.003"
##
## $metric
##                                  crude OR(95%CI) crude P value adj. OR(95%CI)
##                                  NA              NA            NA
## Estimated correlation parameters NA              NA            NA
## No. of clusters                  "39"            NA            NA
## No. of observations              "55"            NA            NA
##                                  adj. P value
##                                  NA
## Estimated correlation parameters NA
## No. of clusters                  NA
## No. of observations              NA
##
## Analysis of 'Wald statistic' Table
##
## Model 1 oneyear_ASR ~ Chemo + PR + vessel + mut_KRAS + CEA_pre + Diameter + margin_best
## Model 2 oneyear_ASR ~ Chemo + PR + vessel + mut_KRAS + CEA_pre + Diameter
##   Df     X2 P(>|Chi|)
## 1  1 8.8074     0.003 **
## ---
## Signif. codes:  0 '***' 0.001 '**' 0.01 '*' 0.05 '.' 0.1 ' ' 1
```

Best cutoff < 5 mm margin (Model D)

```
data$margin_5_best <- data$below5 < asr.roc.tb[asr.roc.tb$below == 5,]$best_threshold

complete_subset <- complete.cases(data[, c("oneyear_ASR", "PR", "vessel", "Diameter",
                                           "CEA_pre", "mut_KRAS", "margin_5_best")])
data.asr.margin5best <- data[complete_subset, ]

asr.gee.5mm <- geeglm(oneyear_ASR ~ Chemo + PR + vessel + mut_KRAS + CEA_pre +
                        Diameter + margin_5_best,
                   id = Patient_ID, corstr = "independence",
                   data = data.asr.margin5best, family = "binomial")

sprintf("N = %d/%d", sum(complete_subset), length(complete_subset))
```

```
## [1] "N = 55/168"
```

```r
geeglm.display(asr.gee.5mm)
```

```
## $caption
## [1] "GEE(binomial) predicting oneyear_ASR by Chemo, PR, vessel, mut_KRAS, CEA_pre, Diameter, margin_5
##
## $table
##                    crude OR(95%CI)     crude P value adj. OR(95%CI)
## ChemoTRUE          "1.65 (0.24,11.61)" "0.613"       "4.56 (0.49,42.72)"
## PRTRUE             "1.31 (0.11,16.11)" "0.832"       "0.25 (0.01,4.5)"
## vesselTRUE         "2.88 (0.77,10.84)" "0.118"       "4.33 (0.44,42.59)"
## mut_KRASTRUE       "0.67 (0.11,4.06)"  "0.665"       "0.58 (0.06,6.01)"
## CEA_pre            "0.99 (0.92,1.08)"  "0.902"       "0.99 (0.88,1.12)"
## Diameter           "1.14 (1.03,1.26)"  "0.01"        "1.33 (0.99,1.79)"
## margin_5_bestTRUE "0 (0,0)"            "< 0.001"     "0 (0,0)"
##                    adj. P value
## ChemoTRUE          "0.183"
## PRTRUE             "0.347"
## vesselTRUE         "0.209"
## mut_KRASTRUE       "0.651"
## CEA_pre            "0.88"
## Diameter           "0.059"
## margin_5_bestTRUE "< 0.001"
##
## $metric
##                                   crude OR(95%CI) crude P value adj. OR(95%CI)
##                                   NA              NA            NA
## Estimated correlation parameters NA              NA            NA
## No. of clusters                   "39"            NA            NA
## No. of observations               "55"            NA            NA
##                                   adj. P value
##                                   NA
## Estimated correlation parameters NA
## No. of clusters                   NA
## No. of observations               NA
```

```r
table(data.asr.margin5best$margin_5_best, data.asr.margin5best$oneyear_ASR)
```

```
##
##         FALSE TRUE
##   FALSE    29    9
##   TRUE     17    0
```

Interpretation: Now the model gets into trouble because there are no ASRs in after ablation where margin < 5 mm $< 45\%$. You can turn it around and say if $< 45\%$ of surface are covered with less than 5 mm the odds of recurrence are 0…

Univariate analysis

```r
geeglm.display(geeglm(oneyear_ASR ~ Chemo, data = data, family = binomial,
                      id = Patient_ID, corstr = "independence"))
```

```
## $caption
## [1] "GEE(binomial) predicting oneyear_ASR by Chemo - Group Patient_ID"
##
## $table
##           OR(95%CI)          P value
## ChemoTRUE "0.61 (0.22,1.69)" "0.344"
##
## $metric
##                                    OR(95%CI) P value
##                                    NA        NA
## Estimated correlation parameters NA        NA
## No. of clusters                    "98"      NA
## No. of observations                "168"     NA
```

```r
geeglm.display(geeglm(oneyear_ASR ~ PR, data = data, family = binomial,
                      id = Patient_ID, corstr = "independence"))
```

```
## $caption
## [1] "GEE(binomial) predicting oneyear_ASR by PR - Group Patient_ID"
##
## $table
##        OR(95%CI)          P value
## PRTRUE "0.35 (0.08,1.62)" "0.181"
##
## $metric
##                                    OR(95%CI) P value
##                                    NA        NA
## Estimated correlation parameters NA        NA
## No. of clusters                    "98"      NA
## No. of observations                "168"     NA
```

```r
geeglm.display(geeglm(oneyear_ASR ~ vessel, data = data, family = binomial,
                      id = Patient_ID, corstr = "independence"))
```

```
## $caption
## [1] "GEE(binomial) predicting oneyear_ASR by vessel - Group Patient_ID"
##
## $table
##            OR(95%CI)          P value
## vesselTRUE "2.15 (0.81,5.71)" "0.127"
##
## $metric
##                                    OR(95%CI) P value
##                                    NA        NA
## Estimated correlation parameters NA        NA
## No. of clusters                    "98"      NA
## No. of observations                "168"     NA
```

```r
geeglm.display(geeglm(oneyear_ASR ~ mut_KRAS, data = data, family = binomial,
                      id = Patient_ID, corstr = "independence", subset = !is.na(mut_KRAS)))
```

```
## $caption
## [1] "GEE(binomial) predicting oneyear_ASR by mut_KRAS - Group Patient_ID"
##
## $table
##               OR(95%CI)          P value
## mut_KRASTRUE "0.49 (0.18,1.3)" "0.15"
##
## $metric
##                                  OR(95%CI) P value
##                                  NA        NA
## Estimated correlation parameters NA        NA
## No. of clusters                  "89"      NA
## No. of observations              "156"     NA
```

```r
geeglm.display(geeglm(oneyear_ASR ~ CEA_pre, data = data, family = binomial,
                      id = Patient_ID, corstr = "independence", subset = !is.na(CEA_pre)))
```

```
## $caption
## [1] "GEE(binomial) predicting oneyear_ASR by CEA_pre - Group Patient_ID"
##
## $table
##          OR(95%CI)       P value
## CEA_pre "1.01 (1,1.02)" "0.16"
##
## $metric
##                                  OR(95%CI) P value
##                                  NA        NA
## Estimated correlation parameters NA        NA
## No. of clusters                  "93"      NA
## No. of observations              "161"     NA
```

```r
geeglm.display(geeglm(oneyear_ASR ~ Diameter, data = data, family = binomial,
                      id = Patient_ID, corstr = "independence", subset = !is.na(Diameter)))
```

```
## $caption
## [1] "GEE(binomial) predicting oneyear_ASR by Diameter - Group Patient_ID"
##
## $table
##           OR(95%CI)          P value
## Diameter "1.09 (1.04,1.14)" "< 0.001"
##
## $metric
##                                  OR(95%CI) P value
##                                  NA        NA
## Estimated correlation parameters NA        NA
## No. of clusters                  "98"      NA
## No. of observations              "168"     NA
```

```r
geeglm.display(geeglm(oneyear_ASR ~ margin_best, data = data, family = binomial,
                      id = Patient_ID, corstr = "independence", subset = !is.na(margin_best)))
```

```
## $caption
## [1] "GEE(binomial) predicting oneyear_ASR by margin_best - Group Patient_ID"
##
## $table
##                   OR(95%CI)            P value
## margin_bestTRUE "13.71 (2.57,73.1)" "0.002"
##
## $metric
##                                  OR(95%CI) P value
##                                  NA        NA
## Estimated correlation parameters NA        NA
## No. of clusters                  "47"      NA
## No. of observations              "65"      NA
```

```r
geeglm.display(geeglm(oneyear_ASR ~ margin_min, data = data, family = binomial,
                      id = Patient_ID, corstr = "independence", subset = !is.na(margin_min)))
```

```
## $caption
## [1] "GEE(binomial) predicting oneyear_ASR by margin_min - Group Patient_ID"
##
## $table
##              OR(95%CI)          P value
## margin_min "0.6 (0.38,0.93)" "0.023"
##
## $metric
##                                  OR(95%CI) P value
##                                  NA        NA
## Estimated correlation parameters NA        NA
## No. of clusters                  "47"      NA
## No. of observations              "65"      NA
```

```r
geeglm.display(geeglm(oneyear_ASR ~ below5, data = data, family = binomial,
                      id = Patient_ID, corstr = "independence", subset = !is.na(below5)))
```

```
## $caption
## [1] "GEE(binomial) predicting oneyear_ASR by below5 - Group Patient_ID"
##
## $table
##        OR(95%CI)               P value
## below5 "23.79 (1.52,372.02)" "0.024"
##
## $metric
##                                  OR(95%CI) P value
##                                  NA        NA
## Estimated correlation parameters NA        NA
## No. of clusters                  "47"      NA
## No. of observations              "65"      NA
```

Interpretation: From the univariate analysis, Diameter and ablation margins are significant factors.
